# MaxComp: Predicting single-cell chromatin compartments from 3D chromosome structures

**DOI:** 10.1371/journal.pcbi.1013114

**Published:** 2025-05-23

**Authors:** Yuxiang Zhan, Francesco Musella, Frank Alber

**Affiliations:** 1 Department of Microbiology, Immunology, and Molecular Genetics, University of California Los Angeles, Los Angeles, California, United States of America; 2 Institute of Quantitative and Computational Biosciences, University of California Los Angeles, Los Angeles, California, United States of America; 3 Department of Quantitative and Computational Biology, University of Southern California, Los Angeles, California, United States of America; Rutgers University: Rutgers The State University of New Jersey, UNITED STATES OF AMERICA

## Abstract

The genome is organized into distinct chromatin compartments with at least two main classes, a transcriptionally active **A** and an inactive **B** compartment, broadly corresponding to euchromatin and heterochromatin. Chromatin regions within the same compartment preferentially interact with each other over regions in the opposite compartment. **A/B** compartments are traditionally identified from ensemble Hi-C contact frequency matrices using principal component analysis of their covariance matrices. However, defining compartments at the single-cell level from sparse single-cell Hi-C data is challenging, especially since homologous copies are often not resolved. To address this, we present MaxComp, an unsupervised method, for inferring single-cell **A/B** compartments based on 3D geometric considerations in single-cell chromosome structures—derived either from multiplexed FISH-omics imaging or 3D structure models derived from Hi-C data. By representing each 3D chromosome structure as an undirected graph with edge-weights encoding structural information, MaxComp reformulates compartment prediction as a variant of the Max-cut problem, solved using semidefinite graph programming (SPD) to optimally partition the graph into two structural compartments. Our results show that the population average of MaxComp single-cell compartment annotations closely matches those derived from ensemble Hi-C principal component analysis, demonstrating that compartmentalization can be recovered from geometric principles alone, using only the 3D coordinates and nuclear microenvironment of chromatin regions. Our approach reveals widespread cell-to-cell variability in compartment organization, with substantial heterogeneity across genomic loci. When applied to multiplexed FISH imaging data, MaxComp also uncovers relationships between compartment annotations and transcriptional activity at the single-cell level. In summary, MaxComp offers a new framework for understanding chromatin compartmentalization in single cells, connecting 3D genome architecture, and transcriptional activity with the cell-to-cell variations of chromatin compartments.

## Author summary

Chromosome conformation capture and imaging techniques have revealed that the genome spatially segregates into at least two functional compartments. Ensemble Hi-C contact frequency maps display checkerboard-like patterns, indicating that chromatin regions fall into two major compartments—likely a result of phase separation—where regions within the same compartment preferentially interact, often over long genomic distances, while interactions with the opposite compartment are minimized. Principal component analysis (PCA) of ensemble Hi-C data is commonly used to identify these compartments. However, because the compartment annotations are derived from a cell population, this method cannot provide information about compartments in single cells. In this study, we introduce an unsupervised graph-based method to predict **A**/**B** compartments in single cells, which utilizes only structural information in single cells. Our results demonstrate that ensemble PCA-based compartment annotations can be reproduced as population averages of our single-cell predictions. Our results reveal substantial cell-to-cell heterogeneity in compartmentalization, with notable variability across genomic regions. Applying our method to multiplexed FISH tracing data links single-cell compartment annotations with gene transcriptional activity, and enables exploration of how local chromatin structure relates to compartment identity. Compared to existing methods, our approach achieves superior compartmentalization scores, offering a robust and interpretable framework for analyzing genome architecture in single cells.

## Introduction

The development of chromosome conformation caption [1–3] and imaging techniques [4–8] has greatly enhanced our understanding of local chromatin folding and higher-order organization across scales, from nucleosomes to chromosome territories. Hi-C studies have revealed key structural features, such as chromatin loops and topological associating domains (TADs) [9–13]. Moreover, chromatin segregates into at least two major compartments, likely driven by protein- and nucleic acid-mediated phase separation [14–16]. A widely used approach to determine chromatin compartmentalization involves applying principal component analysis to the correlation matrix derived from ensemble Hi-C contact frequency matrices [1,2]. The first principal component typically distinguishes **A** and **B** compartments: chromatin regions with positive eigenvalues (**A** compartment) are associated with open, transcriptionally active chromatin, while those with negative eigenvalues (**B** compartment) correspond to more condensed, transcriptionally inactive heterochromatin. These **A**/**B** chromosome compartment profiles correlate strongly with other markers of chromatin state, including histone modification patterns from ChiP-seq data [17] and genomic features such as CG content and CpG density [18].

Most current analysis of chromatin compartments rely on ensemble datasets, describing chromatin properties averaged over a large population of cells. With the development of single-cell genomic technologies such as single-cell Hi-C [19–22] and multiplexed FISH-based chromosome tracing techniques [4–8], it has become increasingly important to characterize chromatin compartments at the single-cell level. Applying ensemble level compartment annotations to single-cell structures is problematic due to substantial structural variability between individual cells. Therefore, methods that infer chromatin compartments from single-cell information are essential for uncovering the relationship between genome structure and gene function at the individual cell level. The approaches can also identify genomic regions with high cell-to-cell variability in compartmental states.

To address this challenge, several approaches have been developed that infer **A**/**B** compartments from either single-cell Hi-C contact [22–24] or distance matrices derived from single-cell chromatin structures [16,22]. For instance, deep learning-based approaches like scGHOST have been proposed to learn **A**/**B** compartment patterns from labeled training data in single-cell Hi-C maps [24]. Other strategies rely on features such as local CG content of a chromatin region for single-cell compartment annotations [22]. However, these approaches often require either high-quality datasets or ground truth annotations for supervised learning, limiting their general applicability.

Here we provide a strategy to determine single cell chromatin compartments based solely on geometric properties of chromosome structures, generated either from computational modeling [25] or super-resolution multiplexed FISH imaging [7,8]. Our approach is based on the assumption that chromosome regions within the same compartment tend to be spatially closer to one another than to regions in opposing compartments. We first embed each chromosome structure into a weighted graph, with nodes representing chromatin regions and edges connecting nodes. Edge weights are derived from geometric features calculated from the spatial distances between the chromatin regions, their sequence distance and their relative geometric locations from nuclear speckles. Compartment segregation is then formulated as a “Max-cut problem”, a graph optimization problem that identifies a division of nodes into two groups such that the sum of weights of edges crossing the cut is maximized [26,27]. We refer to this approach as MaxComp.

The resulting single-cell compartment assignments derived from MaxComp not only conform well with those obtained from ensemble Hi-C matrices but also agree with considerations about structural organization and transcriptional activities at the single-cell level.

We assessed our single-cell compartment predictions by comparing the frequency with which each chromatin region is assigned to the **A** or **B** compartment across the cell population to the first principal component (PC1) values obtained from traditional PCA analysis of ensemble Hi-C data. The strong correlation between these profiles confirms that MaxComp’s single-cell compartment predictions are consistent with established ensemble-level compartment annotations. To further validate our method, we calculated compartmentalization scores, which quantify the structural segregation of compartments. MaxComp consistently achieved the highest scores outperforming other methods, such as Hi-C-based PCA, distance-based PCA using average distance matrices, and a method based on average CG content within spatial neighborhoods. Moreover, our single-cell compartment assignments agree with known properties of active and inactive chromatin, including preferences in nuclear positions, chromatin fiber condensation levels, and proximities to nuclear bodies, which confirmed expected trends in euchromatin and heterochromatin regions. Finally, we assessed single-cell compartment annotations with single-cell transcription data, which confirmed that genes predicted to be in the **A** compartment show a higher transcriptional activity than when the same genes are assigned to the **B** compartment, further supporting the functional relevance of our predictions.

Our results demonstrate that single-cell compartments can be inferred from geometric features of single-cell chromosome structures. The MaxComp approach is robust and can be successfully applied even on chromosome structures imaged at low resolution and coverage. Notably, MaxComp can determine **A** and **B** compartment annotations without relying on statistical models, prior knowledge, or deep learning frameworks, which require large training datasets.

Importantly, we observe substantial cell-to-cell variability in compartment annotations for certain chromatin regions. This highlights the limitations of ensemble-based annotations and underscores the importance of determining chromatin states at the single-cell level to capture biological heterogeneity.

## Results

### Formulating compartmentalization as a Max Cut problem

Our goal is to classify chromatin regions at a defined base-pair resolution (e.g., 200kb) into active (**A**) and inactive (**B**) compartments in single-cell chromosome structures, using only geometric features derived from single-cell data—without relying on ensemble-based compartment annotations from Hi-C data. The assumption is that chromatin within the same compartment shows increased interaction propensity, resulting in overall closer spatial proximity, while chromatin in opposing compartments is more spatially separated. We also expect that chromatin is more likely surrounded by neighboring regions of the same compartment in 3D space, reflecting spatial segregation between **A** and **B** compartments.

**Fig 1:**
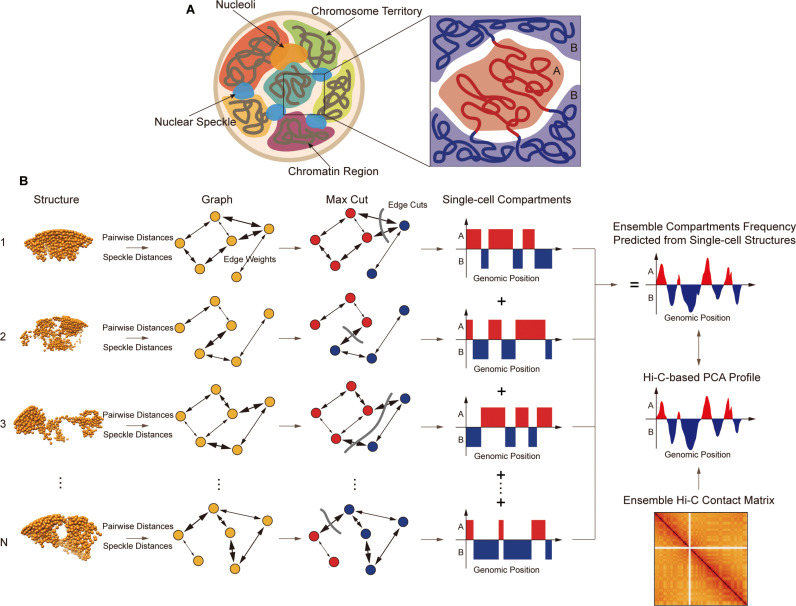
Overview of the MaxComp algorithm working on single-cell structures (A) The assumption of chromatin compartmentalization is based on two parts: Firstly, chromatin regions belong to the same compartment have higher contacts than those from the different compartment; Secondly, chromatin regions of compartment **A** are spatially closer to nuclear speckles than regions of compartment **B**. (B) Every single-cell structure is transformed to an undirected graph which can be represented by an adjacency matrix whose edge weights are decided by its pairwise distances and speckle distances. Max Cut is then applied to the matrix to generate two partitions of nodes which are the prediction of the single-cell compartments of the structure. Ensemble compartment frequency can be calculated by combining a population of single-cell profiles.

Moreover, locations within the nuclear environment can also be indicative of chromatin compartments. For instance, nuclear speckles—interchromatin granule clusters enriched in pre-mRNA splicing factors—are known to be associated with actively transcribed genes. Studies have shown that chromatin near nuclear speckles often harbors highly expressed genes [28]. Therefore, we expect regions of the **A** compartment to localize more frequently in the vicinity of nuclear speckles than regions of the inactive **B** compartment [29,30] (Fig 1A).

To achieve our goal, we represent each single-cell 3D chromosome structure (either from structure models or imaging experiments) as an undirected graph  G=(V, E) where each chromatin region is a node V connected by edges with weights that encode information about their 3D distance and differences in their nuclear microenvironment. Specifically, edge weights between genomic regions i and j are derived from their spatial distance normalized by the expected distance given their genomic separation, along with the z-score difference in their distances to the nearest nuclear speckle (Methods). Our objective is then to partition this graph into two subgraphs in a way that maximizes the total weight of edges between them. Nodes within the same subgraph are then assigned to the same compartment -**A** or **B**-based on their average distance to speckle. This task can be achieved by solving a graph theory problem named the maximum-cut problem (Max-cut), which is NP-hard [31,32]. By solving the Max-cut problem for a given single-cell structure graph, we assign **A/B** compartment annotations to all chromatin regions. Each chromosome structure is then characterized by a single-cell compartment profile, resulting in a compartment profile that captures compartmentalization at the single-cell level.

### Relaxation and approximation of the Max-cut algorithm in MaxComp

We formulate the prediction of chromatin compartments in single cells as a Max-cut problem, which aims to divide a graph into two subgraphs such that the total weight of edges between them is maximized. However, the Max-cut problem is NP-hard, and cannot be solved exactly by a polynomial-time algorithm [31]. Besides greedy approaches, multiple approximation algorithms have been developed to reach a relatively high approximation ratio. Goemans and Williamson [26,28] have proven that certain relaxation and random projection techniques can increase the approximation ratio to about 0.878 to find a near optimal solution for each given Max-cut problem in polynomial time. The relaxation converts the original quadratic programming problem—where each node is represented by an indicator for its compartment type—into a vector programming problem, where nodes are represented by vectors. This transformed problem can be reformulated as a semidefinite programming (SPD) problem, which aims to optimize a linear function subject to positive semidefinite constraints. In our framework, given the Laplacian matrix L, which is a matrix representation of the graph G representing the target chromosome structure (Methods), the goal is to find a symmetric and positive semidefinite matrix A containing information of node labels with diagonal elements set to one so that we can maximize the objective function $\frac{1}{4}tr(L^{T}A)$ (Methods). We demonstrate that maximizing intra-compartment similarity while simultaneously minimizing similarity between compartments can be accomplished with the same objective function. This confirms that single-cell compartment annotations are uniquely determined by L, which encodes both spatial geometry and nuclear context (Methods).

Ideally, we aim to obtain a strictly positive semidefinite matrix A, enabling Cholesky decomposition to generate $A={VV}^{T}$, where V contains row vectors representing nodes (i.e., genomic regions) in the hyperspace. However, generating a strictly semidefinite (SD) matrix requires lengthy computation times due to small convergence thresholds, especially on large graphs with hundreds of nodes. Therefore, we employ an approximation strategy to generate a close SD matrix with a larger convergence threshold. Subsequently, we apply lower-diagonal-lower (LDL) decomposition, a variant of Cholesky decomposition, which decomposes the target matrix into two triangular matrices and a diagonal matrix to obtain an approximated SD matrix $A^{\prime }=V^{\prime }{V^{\prime }}^{T}$, facilitating the discovery of approximated row vectors (Methods). We prove that the difference in Euclidean norm between matrix $V^{\prime }$ containing approximated row vectors and matrix V is strictly governed by the difference in Euclidean norm between approximated matrix $A^{\prime }$ and matrix A, ensuring minimal errors during approximation (Methods). Furthermore, Goemans and Williamson [26,27] introduced a random projection approach to iteratively generate hyperplanes, dividing all row vectors of $V^{\prime }$ into two groups labeled as compartment **A** and **B**. Using the adapted version of this algorithm, we are able to perform MaxComp several times faster, which is particularly suitable for high-resolution chromosome structures with several hundreds of chromatin regions.

### MaxComp prediction of single cell compartments from 3D chromosome structures

We apply our MaxComp approach to three different datasets. First, we use single-cell 3D genome structures generated by the integrative genome modeling (IGM) platform [25], using Hi-C [3], Lamin B1 DamID [33] and SPRITE [34] data as input information [25]. These structures are resolved at 200kb base-pair resolution and also predict the spatial distance of each chromatin region to the nearest nuclear speckle in each single cell, using a Markov clustering approach as described in [30]. Second, we apply MaxComp to 3D genome structures of human IRM90 cells from DNA multiplexed error-robust fluorescence in situ hybridization (MERFISH) experiments, a chromosome tracing method that images 3D genome structures for more than 7,000 single cells at a coverage of around 3Mb [7]. Thus, 3D coordinates are available for chromatin regions spaced approximately every 3Mb along the linear genome, providing their spatial positions within the nucleus. The method also imaged the locations of nuclear bodies within the same cell, allowing to estimate also the spatial distance of each chromatin region to nuclear bodies. Finally, we also apply our method to chromosome structures from DNA sequential fluorescence in situ hybridization (seqFISH+) imaging [8], which traces mouse embryonic stem cell (mESC) chromosomes at 1Mb coverage for 444 imaged cells (888 chromosome copies) and also provides relative locations of nuclear speckles within the same imaged cells.

### Applying MaxComp to chromosome structures from IGM genome structure modeling

**Fig 2:**
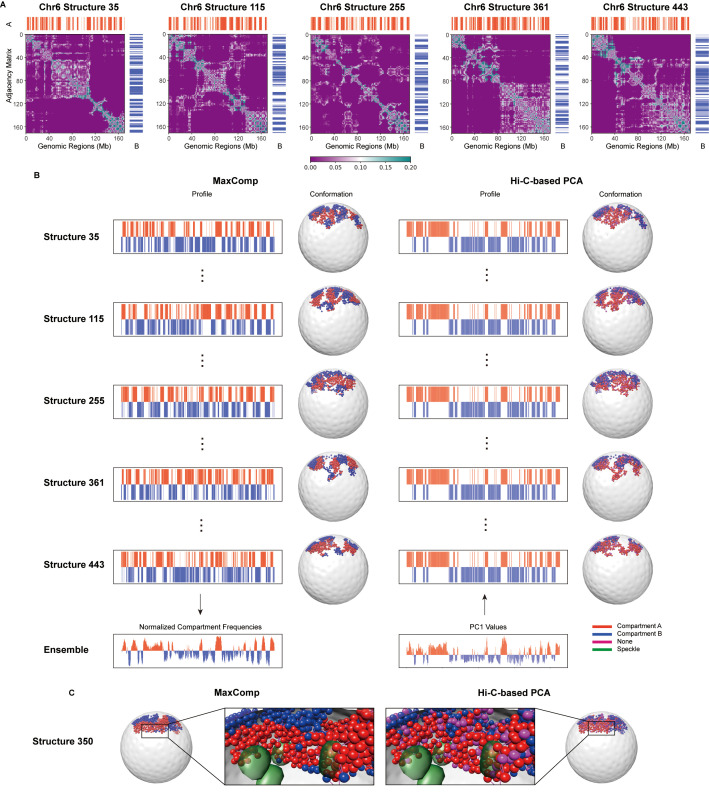
Selected example of model compartments predicted by MaxComp and the corresponding structures of H1-hESC Chr6 (A) The predicted compartments for structure 35, 115, 255, 361, 443 of H1-hESC Chr6 together with the input adjacency matrices of the MaxComp approach. (B) The compartment profile and 3D visualization of structure 35, 115, 255, 361, 443 of H1-hESC Chr6 colored by compartments (red in compartment **A** and blue in compartment **B**) from both experiment and the MaxComp prediction showed together with the nucleus envelope. (C) Predicted speckles (green) showed together with the single-cell examples colored compartments labeled by MaxComp and the Hi-C-based PCA.

First, we apply MaxComp on chromosome structures extracted from whole genome structures of H1-hESC cells generated by integrative modeling [25]. These structures also predict positions of nuclear bodies, such as nuclear speckles and nucleoli [25,30]. Specifically, we took 500 3D structures of chromosome 6 and 500 structures of chromosome 10 from single-cell H1-hESC whole genome structures. We then applied MaxComp to generate a single-cell compartment profile vector for each chromosome structure (Methods) (Fig 1B). The input graph of a structure can be represented by an adjacency matrix, where the entry in row i and column j represents the weight of the edge connecting vertex i and vertex j. Adjacency matrices and predicted single-cell compartment profile vectors vary considerably across individual structures (Fig 2A and Fig 2B, left column). Noticeably, along the chromosome sequence, single-cell compartment annotations show more frequent transitions between **A** and **B** compartments (Fig 2B, left column) compared to the patterns derived from ensemble Hi-C derived (Fig 2B, right column). These transitions vary between individual cells, reflecting underlying structural heterogeneity. Despite this variability along the linear genome, visualization of single-cell compartments in 3D structures reveals pronounced spatial segregation of **A**/**B** compartments, with extended clusters of chromatin regions belonging to the same compartment forming distinct spatial domains (3D structures in Fig 2B,C, left panels).

### MaxComp-predicted single-cell compartments reconstitute ensemble Hi-C compartment profiles

**Fig 3:**
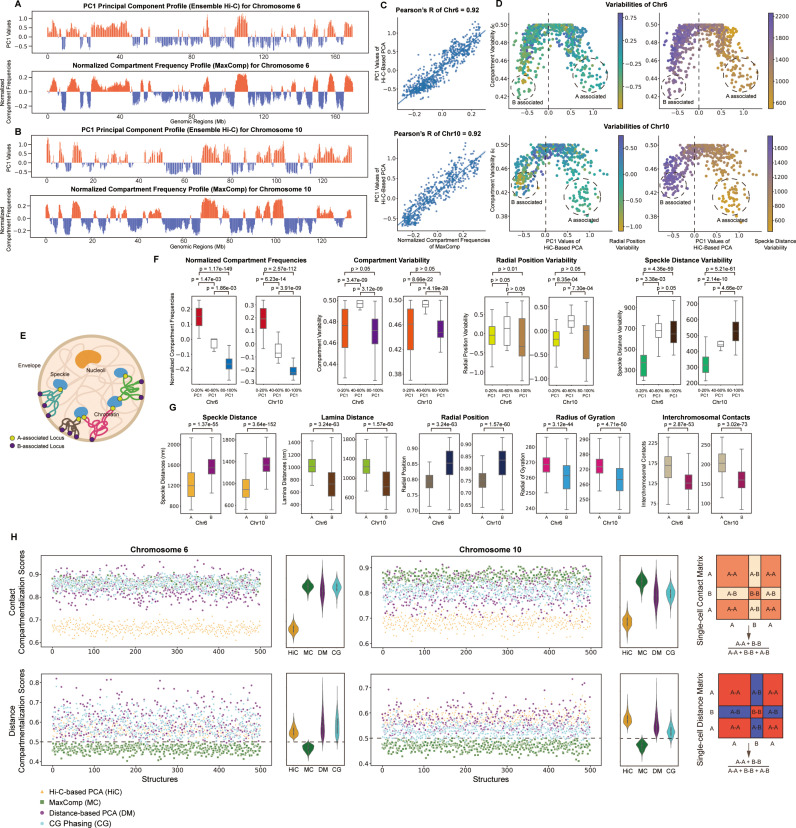
Prediction of model compartments by MaxComp and its comparison with other methods on H1-hESC Chr6 and Chr10 (A) The experimental profile obtained from the Hi-C-based principal component analysis and the compartment profile predicted by MaxComp on 500 modeled structures of H1-hESC Chr6. (B) The experimental profile obtained from the Hi-C-based principal component analysis and the compartment profile predicted by MaxComp on 500 modeled structures of H1-hESC Chr10. (C) Scatter plot between the normalized compartment frequencies of MaxComp and the PC1 values together with the Pearson’s correlation coefficient between the two samples on each chromosome. (D) Scatter plots of MaxComp single-cell compartment variabilities against PC1 values from the Hi-C-based PCA annotations for each chromosome. Each dot represents a genomic region colored by its value of cell-to-cell radial position variability in their radial positions (Left) or cell-to-cell speckle distance variability (Right). (E) Illustration of **A**-associated locus and **B**-associated locus acting as anchors at speckles and envelope during chromatin folding. (F) Comparison of compartment profile (p-value = 8.84e-139 and 4.74e-140), compartment variability (p-value>0.05), radial position variability (p-value>0.01) and speckle distance variability (p-value = 4.36e-59 and 5.21e-61) between top 20% PC1 locus and bottom 20% PC1 locus of H1-hESC Chr6 and Chr10. (G) Comparison of speckle distances (p-value = 1.37e-55 and 3.64e-152), lamina distances (p-value = 3.24e-63 and 1.57e-60), radial position (p-value = 3.24e-63 and 1.57e-60), radius of gyration (p-value = 3.12e-44 and 4.71e-50) and interchromosomal contacts (p-value = 2.87e-53 and 3.02e-73) between compartment **A** beads and compartment **B** beads on 500 structures of H1-hESC Chr6 and Chr10. (H) Comparison of compartmentalization scores between the Hi-C-based PCA annotation, the MaxComp prediction, the distance-based PCA annotation and the CG phasing prediction for each structure from H1-hESC Chr6 and Chr10 showed together with the corresponding violin plots and illustration.

We first validate our MaxComp predictions by comparing the compartment frequency of each chromatin region across cells (Methods) with the PC1 values obtained from ensemble Hi-C matrices through principal component analysis (Fig 3A). The absolute value of a chromatin region’s compartment frequency of a given chromatin region reflects the fraction of times it is consistently assigned to either the **A** or **B** compartment across all single cells (Methods). We observed high correlations (Pearson ’s correlation >= 0.9) between predicted normalized compartment frequencies and PC1 values for all studied chromosomes (Fig 3A,B,C). These results demonstrate that population-averaged single-cell chromosome compartment annotations from MaxComp closely reconstitute ensemble-level compartment profiles derived from Hi-C, providing independent validation [1,3]. Importantly, this agreement arises despite MaxComp relying solely on geometric features of 3D structures, without using ensemble Hi-C contact data or PCA (Fig 2B).

We observe that chromatin regions with intermediate ensemble Hi-C PC1 values (40–60 percentile; i.e., middle PC1 quintiles) show relatively low absolute compartment frequencies (close to 0) and thus show a significantly higher variability ${\delta c}_{i}$ in their compartment assignments across cells (Methods) (Fig 3D left panel, Fig 3E) than regions with large absolute PC1 values (p-value = 3.47e-09 and 3.12e-09 for Chr6 and p-value = 8.66e-22 and 4.19e-28 for Chr10 Fig 3F). Notably, these regions with intermediate PC1 values also show significantly greater variability in their radial positions in the nucleus than regions with high absolute PC1 values (p-value = 8.35e-04 and 7.30e-04 for Chr10, Fig 3D radial position variability panel). These observations suggest that such regions may have higher transcriptional heterogeneity between cells than regions with consistently high absolute PC1 values, which tend to have the same dominant compartment assignments in the large majority of cells [30]. However, this remains a speculative interpretation at this point.

Therefore, regions with the highest and lowest PC1 value quintiles show the highest absolute compartment frequencies (Fig 3D, left panels) and thus, the lowest compartment variabilities between cells than regions with intermediate PC1 value quintiles (p-value = 1.47e-03 and 1.86e-03 for Chr6 and p-value = 6.23e-14 and 3.91e-09 for Chr10, Compartment Variability panel in Fig 3F) (Fig 3D, left panels). These regions show also lower variability in their radial positions in the nucleus between cells and tend to be located either at the nuclear exterior lamina compartment or in the nuclear interior close to nuclear speckles—supporting previous findings that such regions serve as structural anchors for genome organization [30] (Fig 3D) (radial position variability panel in Fig 3F). For instance, regions with highest normalized **A**-compartment frequencies show generally lower speckle distance variability and high affinity to nuclear speckles (Fig 3D) (speckle distance variability panel in Fig 3F).

Next, we evaluate the robustness of the MaxComp pipeline across varying population sizes ranging from 10 to 500 single-cell chromosome structures. We found that as few as 200 structures are sufficient to yield a Pearson’s correlation of at least 0.90 between MaxComp-predicted normalized compartment frequency profiles and ensemble Hi-C-derived compartment profiles (**S2**(ABC) Fig). Even with only 10 structures the Pearson’s correlation remains moderate at 0.64 (**S2**(ABC) Fig). However, larger population sizes show the best performance and overall smoother normalized average compartment frequency profiles.

In summary, these results demonstrate that single-cell compartments can be determined from 3D structural information alone, and that MaxComp performs robustly across different chromosomes.

### Single-cell compartment predictions are consistent with expected chromatin structure properties

We further assess our single-cell compartment predictions by measuring several structural properties of chromatin with predicted **A** and **B** compartments, including nuclear radial positions, chromatin fiber condensation, and distances to nuclear bodies.

Our analysis confirms several expected trends for euchromatic **A** and heterochromatin **B** compartment chromatin: First, we find chromatin predicted in **A** compartment to have significantly larger radius of gyration (p-value = 3.12e-44 for Chr6 and 4.71e-50 for Chr10), meaning that these regions are less condensed than **B** compartment chromatin (Radius of gyration panel in Fig 3G). Also, compartment **A** regions have a significantly higher number of interchromosomal contacts in single cells than compartment **B** regions (p-value = 2.87e-53 for Chr6 and 3.02e-73 for Chr10). Thus, **A** compartment chromatin in single cells is more frequently located at the exterior of chromosome territories (Fig 3G).

Also, chromatin in the **A** compartment are located more interior in the nucleus with smaller radial positions than those in the **B** compartment (p-value <= 1.57e-60 for Chr6,10) and subsequently smaller speckle distances (p-value <= 1.37e-55 for Chr6,10) (Fig 3G).

These trends hold across additional analyzed chromosomes (e.g., Chr8, Chr12, Chr15, Chr18), which show very similar results and maintain high concordance with Hi-C-based PC1 profiles (Pearson’s correlation >= 0.9 between predicted chromosome frequencies and Hi-C based PC1 profiles) (S3(ABC) Fig and S4(ABC) Fig).

### Single-cell compartments show high compartmentalization scores

To further validate our results, we calculate a compartmentalization score based on chromatin-chromatin contacts in single cell structures. The contact-compartmentalization score (CCS) is defined as defined as the fraction of chromatin-chromatin contacts that occur within the same compartment (intra-compartment contacts), relative to the total number of contacts regardless of compartment assignment (Methods) (Fig 3H). This value is averaged over all single-cell chromosome structures. A higher CCS score indicates a stronger spatial segregation between **A** and **B** compartments, representing a more favorable and well-defined compartment state. We compare the contact compartmentalization scores from MaxComp with three alternative methods, namely the aforementioned ensemble Hi-C-based PCA analysis [2,3,8], a distance-based PCA analysis [4,35] and CG phasing [22] (Methods). The distance-based PCA method (DM) uses the average distance matrix calculated from all single-cell chromosome structures and applies principal component analysis to define **A**/**B** compartment annotations. Similar to Hi-C-based PCA analysis, the resulting compartment annotations are then assigned uniformly across all single-cell chromosome structures. The CG phasing method assigns a target region to the **A** compartment based on the total amount of CG DNA content from all chromatin regions within its 3D spatial neighborhood.

MaxComp predictions achieve the highest average CCS compartment score among the three methods, outperforming ensemble Hi-C-based PCA (HiC) and distance-based PCA (DM) compartment predictions (Fig 3H). Additionally, we also tested a compartment score based on distances rather than of chromatin-chromatin contacts. The distance-compartmentalization score (DCS) is defined as the ratio of the average distances between chromatin regions within the same compartment and the average distances between all chromatin regions, regardless of their compartment annotations (Methods) (Fig 3H). Here, a smaller DCS score indicates a more favorable spatial compartment segregation. Our results show that MaxComp compartment annotations produce a better spatial segregation between the two compartments at single-cell level, as evidenced by the smallest DCS score compared to the other methods (Fig 3H).

### Applying MaxComp to multiplexed FISH imaging datasets reveals the relationship between single-cell compartments and transcription signals

Next, we evaluate MaxComp on chromosome structures imaged by integrated multiplexed FISH experiments [7,8]. These chromosome tracing experiments provide chromosome structures at considerably sparser coverage. For instance, chromosomes of human IMR90 cells are imaged at 3Mb step size by DNA MERFISH [7], while chromosomes in mESC cells are imaged at 1Mb step size in DNAseqFISH+ [8]. By combining multiplexed FISH chromosome tracing with immunofluorescence imaging these methods can also detect the locations of nuclear speckles and nucleoli in the same cells. Moreover, the datasets are also integrated with RNA-MERFISH and RNAseqFISH + , providing information about active transcription of specific genes in the same imaged cells [7,8].

**Fig 4:**
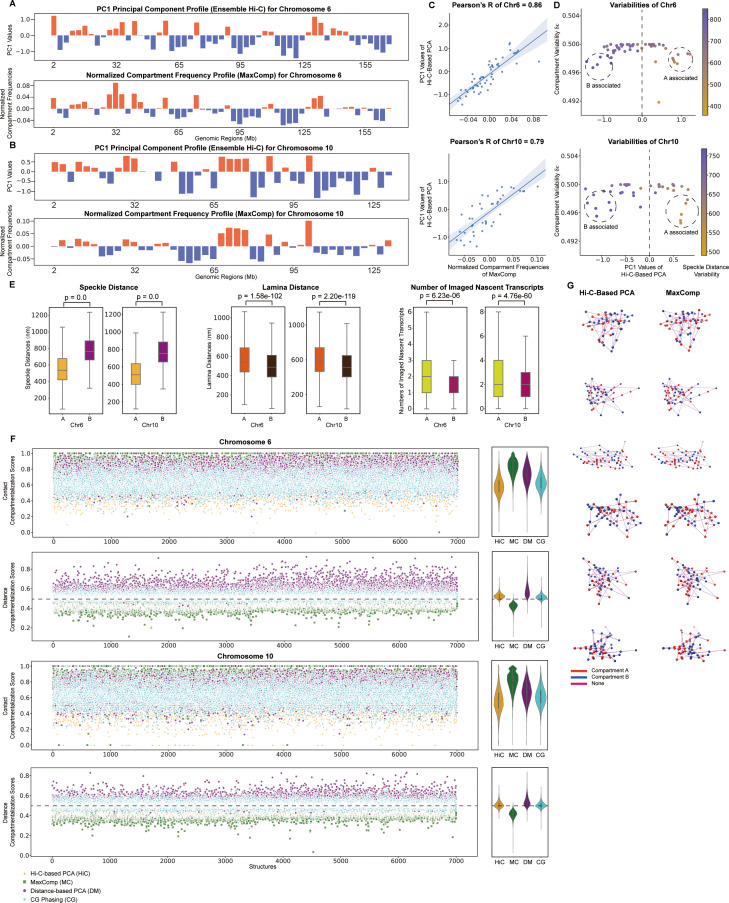
Prediction of DNA-MERFISH compartments by MaxComp and its comparison with other methods on IMR90 Chr6 and Chr10 (A) The experimental profile obtained from the Hi-C-based principal component analysis and the compartment profile predicted by MaxComp on 7,000 DNA-MERFISH structures [7] of IMR90 Chr6. (B) The experimental profile obtained from the Hi-C-based principal component analysis and the compartment profile predicted by MaxComp on 7,000 DNA-MERFISH structures of IMR90 Chr10. (C) Scatter plot between the normalized compartment frequencies of MaxComp and the PC1 values together with the Pearson’s correlation coefficient between the two samples on each chromosome. (D) Scatter plot of compartment variabilities against PC1 values from the Hi-C-based PCA annotations on each chromosome. Each dot represents a genomic region colored by its value of speckle distance variability. (E) Comparison of speckle distances (p-value = 0.0 and 0.0), lamina distances (p-value = 1.58e-102 and 2.20e-119) and numbers of imaged nascent transcripts (p-value = 6.23e-06 and 4.76e-60) between compartment **A** beads and compartment **B** beads on 7,000 DNA-MERFISH structures of IMR90 Chr6 and Chr10. (F) Comparison of compartmentalization scores between the Hi-C-based PCA annotation, the MaxComp prediction, the distance-based PCA annotation and the CG phasing prediction for each structure on IMR90 Chr6 and Chr10 showed together with the corresponding violin plots. (G) The 3D visualization of 6 selected DNA-MERFISH structures of IMR90 Chr6 colored by compartments (red in compartment A and blue in compartment B) from both ensemble PC1 and Max-cut prediction.

We tested our method to 7,000 structures of chromosome 6 and chromosome 10 from IMR90 cells, imaged using DNA MERFISH [7]. Chromosome 6 is represented by a total of 55 imaged loci. Despite the relatively sparse coverage, MaxComp performs well in predicting **A**/**B** compartment annotations. For instance, the averaged single-cell normalized compartment frequency profiles predicted by MaxComp show high correlations with the ensemble Hi-C-based PCA compartment profiles (Pearson ’s correlation >= 0.8) (Fig 4A,B,C). We also observe lower compartment variability for chromatin regions with high or low PC1 values, derived from independent Hi-C data analysis [2,3,8] (Fig 4D). Also, **A** compartment chromatin shows significantly smaller speckle distances (p-value = 0.0 for Chr6 and 0.0 for Chr10) and larger distances to the nuclear lamina (p-value = 1.58e-102 for Chr6 and 2.20e-119 for Chr10) compared to **B** compartment chromatin (Fig 4E).

A benefit of chromosome tracing by DNA MERFISH is the ability to concurrently measure nascent gene transcription for a selected group of genes in the same cell by RNA MERFISH imaging [7]. Interestingly, we find for all tested genes a higher nascent transcription signal (i.e., number of imaged nascent transcripts in a cell) in those structures where the gene is predicted to be in the **A** compartment in comparison to cells where the same gene locus is predicted to be in the **B** compartment (p-value = 6.23e-06 for Chr6 and 4.76e-60 for Chr10) (Fig 4E). Overall genes in the **A** compartment are more likely associated with active transcription, supporting studies about the role of nuclear compartmentalization for gene transcription [36]. However, our results also indicate that transcription can also occur for genes in the **B** compartment, in support of recent studies using RD-SPRITE, which simultaneously maps 3D genome structure and nascent RNA transcription genome-wide [37] (S6(A) Fig). Overall, the average transcription profile (i.e., number of imaged transcription spots averaged across the cell population) shows good correlation with the predicted **A**/**B** compartment profile as well as with the gene density profile (S6(BC) Fig). Similar results are also found for other chromosomes, such as chromosome 10 (S7(ABC) Fig).

Finally, we find both compartmentalization scores, CCS and DCS, are significantly better for compartments predicted by MaxComp compared to those predicted by the distance based PCA (DM), single-cell CG phasing method, or ensemble based Hi-C PCA (Hi-C) (**Fig 4F**), confirming our previous observations.

**Fig 5:**
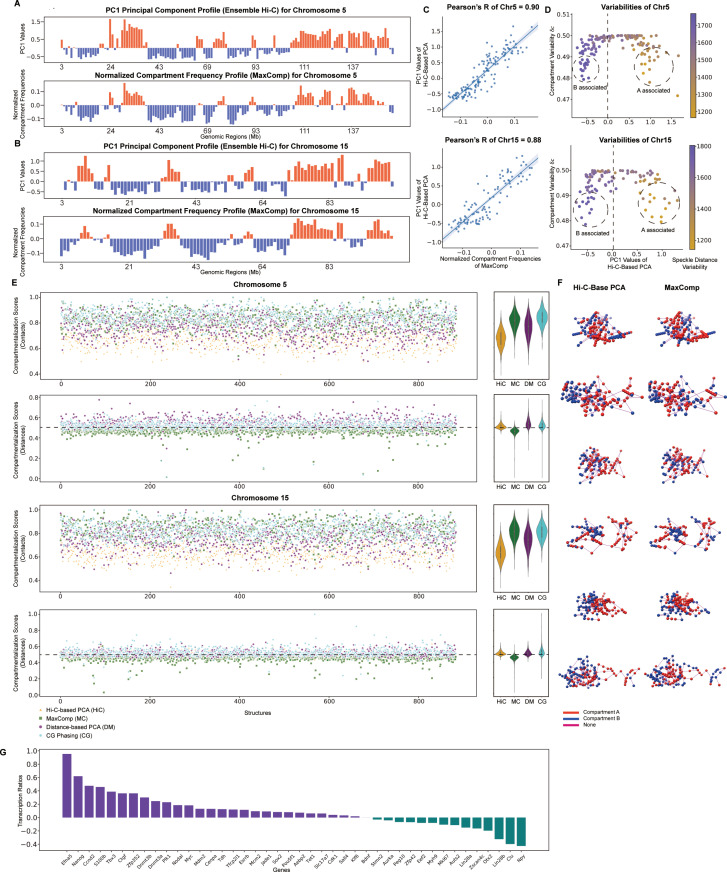
Prediction of SeqFISH **+** **compartments by MaxComp and its comparison with other methods on mESC Chr5 and Chr15** (A) The experimental profile obtained from the Hi-C-based principal component analysis and the compartment profile predicted by MaxComp on 886 SeqFISH+ structures [8] of mESC Chr5. (B) The experimental profile obtained from the Hi-C-based principal component analysis and the compartment profile predicted by MaxComp on 884 SeqFISH+ structures of mESC Chr15. (C) Scatter plot between the normalized compartment frequencies of MaxComp and the PC1 values together with the Pearson’s correlation coefficient between the two samples on each chromosome. (D) Scatter plot of compartment variabilities against PC1 values from the Hi-C-based PCA annotations on each chromosome. Each dot represents a genomic region colored by its value of speckle distance variability. (E) Comparison of compartmentalization scores between the Hi-C-based PCA annotation, the MaxComp prediction, the distance-based PCA annotation and the CG phasing prediction for each structure on mESC Chr5 and Chr15 showed together with the corresponding violin plots. (F) The 3D visualization of 6 selected SeqFISH+ structures of mESC Chr5 colored by compartments (red in compartment **A** and blue in compartment **B**) from both experiment and the MaxComp prediction. (G) Log fold change of the average transcription level (number of mRNA transcript spots detected) of cells with both copies labeled with **A** (**A** cells) against the level of cells with both copies labeled with **B** (**B** cells) for each gene.

Next, we applied MaxComp to chromosome tracing data from DNA SeqFISH+ of the mESC cell line [8]. These structures were imaged genome-wide at 1Mb coverage. Also here, we found similar results with high Pearson’s correlations >= 0.8 between PCA-based compartment profiles from ensemble Hi-C and MaxComp predicted normalized compartment frequency profiles in both studied chromosomes. Also here, the CCS and DCS compartmentalization scores show good spatial segregation for the MaxComp compartments (**Fig 5A,B,C,D,E).** The CG method shows similar performance to MaxComp, although with a lower contact-based compartmentalization score. However, MaxComp performs substantially better when calculating the distance-based compartmentalization score (**Fig 5E**).

We also analyzed transcription data for 42 genes, whose nascent transcription levels have been determined in single cells [8]. To analyze these data, we divide the structures in two groups: first, structures where both homologous gene copies are predicted to be in the **A** compartment (**A** cells) and second, those structures where both gene copies were predicted to be in the **B** compartment (**B** cells). For each of these cells the nascent transcription level (number of mRNA transcript spots detected) for each of the 42 genes was measured by RNAseqFISH+ [8]. For each gene, we then calculated the transcription ratio as the average nascent transcription level for the genes in **A** cells over the average transcription level of the same gene in **B** cells (Methods). We found that 67% of all measured genes have positive transcription ratio, indicating that genes in compartment **A** (**A** cells) are expressed at higher levels in structures where the gene is predicted to be than those in compartment **B** (**B** cells) (**Fig 5G**). Additionally, the distributions of normalized transcription levels for all 42 genes in **A** compartment are statistically significantly different from those in the **B** compartment (paired t-test p-value < 0.05) (S8 Fig).

## Discussion

Experimental evidence from Hi-C and imaging studies shows that chromatin segregates spatially into at least two distinct functional compartments. Compartment **A** is associated with open, transcriptionally active euchromatin while compartment **B** corresponds to more condensed heterochromatin. The spatial segregation manifests in Hi-C contact frequency maps as a checkerboard-like pattern, reflecting preferential interactions of chromatin within the same compartment and reduced interactions between opposing compartments. Principal component analysis of ensemble Hi-C contact frequency maps has been widely used to classify chromatin into **A** and **B** compartments, based on the first principal component (PC1) values. However, because this analysis is based on averaged data across thousands of cells, it cannot reveal compartment information at the single-cell level. To better understand the underlying principles of chromatin compartment formation, it is important to detect compartments at single-cell level, and study if and how chromatin compartments vary between individual cells.

To address this problem, we developed MaxComp, a graph-theory-based method that determines chromatin compartments in single cells using only geometric properties from 3D chromosome structures. By representing each chromosome structure as an undirected graph, MaxComp optimally partitions the graph into two compartment subgraphs, such that the resulting single-cell **A/B** compartment annotations across the cell population yield compartment frequency profiles that closely match those derived from principal component analysis of ensemble Hi-C. Furthermore, chromatin regions assigned to single-cell **A** and **B** compartments exhibit distinct structural characteristics—such as radial positioning and local chromatin condensation (measured by the radius of gyration)—that are consistent with the expected differences between euchromatin and heterochromatin.

We demonstrate that our method is robust across different structural resolutions and can be successfully applied to low-coverage chromosome structures obtained from DNA MERFISH and DNAseqFISH+ tracing experiments. When combined with nascent mRNA imaging, our analysis shows that most genes predicted to be part of the **A** compartment consistently show higher transcriptional activity compared to the same genes located in the **B** compartment. These results demonstrate that single-cell compartment annotations derived solely from geometric features of 3D chromosome structures are sufficient to capture biologically meaningful compartment organization at the single-cell level.

Our analysis shows that the cell-to-cell variability in single-cell compartment annotations is strongly correlated with the ensemble-Hi-C PC1 values. Chromatin regions with high absolute PC1 values show low compartment fluctuations. Instead, chromatin regions with intermediate PC1 values (i.e., small absolute values) show the highest fluctuations in compartment annotations between individual cells. Interestingly, these are also chromatin regions that show relatively high variability in their radial positions between individual cells. However, the high compartment fluctuations of these specific chromatin regions may also indicate a limitation of the two-state compartment definition and that these regions may not be functionally clustered into either **A** or **B** compartments.

Moreover, our single-cell compartment annotations show good performance against other prediction methods, yielding higher compartmentalization scores and thus higher spatial segregation of **A** and **B** compartment chromatin.

Overall, our study emphasizes the importance of defining compartments at single-cell level, which can be achieved by using only geometric considerations in 3D chromosome structures. These structures can be derived from structure modeling or chromosome tracing experiments.

Biologically, our method offers unique opportunities to relate single-cell structural features with distinct functional properties, for instance, enabling more precise identification of transcriptionally active regions in individual cells, not achievable from ensemble-based compartment analysis. Furthermore, our results can provide insights into the heterogeneity of gene expression across single cells, which could play an important role in cell differentiation. Using imaging data in complex tissues, our single-cell compartment predictions may help explore whether compartment states change along continuous spatial trajectories across a tissue.

Importantly, MaxComp is an unsupervised, *de novo* approach that does not rely on pre-trained models or ensemble-based labels and is broadly applicable across chromosome structures with varying coverage and resolution. In the future, higher-resolution structures from imaging are needed to further assess the quality of compartment predictions. Currently, our method relies exclusively on intra-chromosomal distances to infer single-cell compartments, as it is designed to analyze individual chromosomes. Incorporating inter-chromosomal interactions into this framework is a promising direction for future work, with the potential to enhance prediction accuracy and extend the method’s applicability to genome-wide single-cell compartment profiling.

## Materials and methods

### Definition of Max Cut

Given an undirected graph  G=(V, E), with V as vertices of the graph and E as edges connecting vertices. A cut in G is a defined as a subset of the graph S ⊆ V. Let $\overset{―}{S}=V\;\backslash \;S$, then the Max-cut problem is finding the cut S such that $\left|E(S,\;\overset{―}{S})\right|$ the sum of weights of edges connecting set S and set $\overset{―}{S}$ is maximized. Because the Max-cut problem is NP-hard, multiple approximation algorithms exist, including greedy algorithms and local search. The efficiency of an approximation algorithm can be defined by the approximation ratio:


$$r_{A}=\frac{A(Max\;Cut)}{OPT(Max\;Cut)}\;$$

where A(Max Cut) is the result of the approximate algorithm A and OPT(Max Cut) is the optimal result of the problem. Sahni and Gonzalez [31] developed an approximation algorithm achieving $r_{A}=0.5$, while other approximations reach slightly higher approximation ratios [27]. The approximation ratio can be substantially improved by formulating the original problem as a quadratic programming problem [26,27].

### Quadratic programming problem

The relaxation and reformulation of the problem follows the approach by Goemans and Williamson [26,27]. Since graph cutting is equivalent to partitioning its nodes into two groups, we use an indicator variable $x_{i}\;\in \;\{1,\;-1\}$ to represent the group assignment of node i. The objective is to maximize the total weight of edges connecting nodes within both group partitions. This leads to a quadratic programming formulation of the Max-Cut problem:


$$Maximize\;\frac{1}{2}\sum_{(i,\;j)\in E}w_{ij}(1-x_{i}x_{j})$$

$$Subject\;to\;x_{i}\;\in \;\{1,\;-1\}$$

Once the optimal indicators $x_{i}$ are determined, nodes can be partitioned into two groups based on their assignments:

$\begin{array}{c} S=\left\{i:x_{i}=1\right\}\;and\;\overset{―}{S}=\left\{i:x_{i}=-1\right\} \end{array}$

### Vector programming problem

Instead of representing each node with a one-dimensional indicator, we relax the label of node i to a vector $\begin{array}{c} v_{i}\in R^{n} \end{array}$. The Max-Cut problem can then be relaxed as:


$$Maximize\;\frac{1}{2}\sum_{(i,\;j)\in E}w_{ij}(1-{v_{i}}^{T}v_{j})$$

$${{Subject\;to\;v}_{i}}^{T}v_{i}={\left\|v_{i}\right\|}_{2}^{2}=1$$

$$v_{i}\in {\mathbb{R}}^{n}v_{i}\in {\mathbb{R}}^{n}$$

This semidefinite relaxation enables efficient approximation through convex optimization techniques.

### Semidefinite programming problem

In the vector programming formulation, each dot product ${v_{i}}^{T}v_{j}$ can be represented as $a_{ij}$. Defining a matrix $A=(a_{ij})$, we can rewrite the problem as:


$$Maximize\;\frac{1}{2}\sum_{(i,\;j)\in E}w_{ij}\left(1-a_{ij}\right)\;subject\;to\;a_{ii}=1$$

Since $a_{ij}=$ ${v_{i}}^{T}v_{j}$, letting $V={\left(v_{1},\;v_{2},\;\ldots ,{\;v}_{n}\right)}^{T}$, we have $A={VV}^{T}$. The matrix A is symmetric by the commutative properties of the dot product. Moreover, for any nonzero vector $\begin{array}{c} y\in R^{n} \end{array}$,


$$y^{T}Ay=y^{T}{VV}^{T}y={\left(V^{T}y\right)}^{T}\left(V^{T}y\right)={\left\|V^{T}y\right\|}_{2}^{2}\geq 0$$

implying that A is positive semidefinite.

We can reformulate the Max-cut problem as a semidefinite programming (SDP) problem:

$Maximize\frac{1}{4}{\langle L,\;A\rangle }_{F}=\frac{1}{4}tr(L^{T}A)\$Maximize\frac{1}{4}{\langle L,\;A\rangle }_{F}=\frac{1}{4}tr(L^{T}A)$


$$Subject\;to\;diag\left(A\right)=1,\;A≽0,\;A=A^{T}$$

A symmetric and positive semidefinite

where L=E−W is the graph Laplacian matrix, W is the adjacency matrix, E is the degree matrix and ${\langle X,\;Y\rangle }_{F}$ is the Frobenius inner product between matrix X and Y.

### Formulation of the compartment prediction problem

To predict chromatin compartments from single-cell chromosome structures, we aim for chromatin regions (i.e., beads) in compartment **A** to preferentially contact other beads in compartment **A** more frequently, and likewise for beads in compartment **B**. That is, intra-compartment contacts should be maximized, and inter-compartment contacts minimized. We represent each single-cell chromosome structure as a graph where nodes represent beads and edge weights between every two nodes are derived from a combination of their spatial distance and their relative distances from nuclear bodies, capturing aspects of nuclear architecture (see section “Transforming 3D structures to graphs”).

Using a semidefinite programming framework, we want to maximize the sum of weights for inter-compartment edges, which contribute $\frac{1}{2}\sum_{(i,\;j)\in E}w_{ij}(1-a_{ij})$, and minimize the sum of weights of intra-compartment edges connecting nodes from the same compartment, which contribute $\frac{1}{2}\sum_{(i,\;j)\in E}w_{ij}(1+a_{ij})$. Then the original problem can be formulated as:


$$Maximize\;\frac{\lambda_{1}}{2}\sum_{(i,\;j)\in E}w_{ij}(1-a_{ij})-\frac{\lambda_{2}}{2}\sum_{(i,\;j)\in E}w_{ij}\left(1+a_{ij}\right)\;subject\;to\;a_{ii}=1$$

where $\lambda_{1}$ and $\lambda_{2}$ are factors to balance inter-compartment and intra-compartment information with $\lambda_{1}+\lambda_{2}=1$. The programming problem in matrix form can be formulated as:


$$Maximize\;\frac{\lambda_{1}}{4}{\langle L,\;A\rangle }_{F}-\frac{\lambda_{2}}{4}{\langle L,\;2I-A\rangle }_{F}=\frac{\lambda_{1}+\lambda_{2}}{4}{\langle L,\;A\rangle }_{F}-\frac{\lambda_{2}}{2}{\langle L,\;I\rangle }_{F}=\frac{1}{4}{\langle L,\;A\rangle }_{F}-\frac{\lambda_{2}}{2}{\langle L,\;I\rangle }_{F}=\frac{1}{4}tr\left(L^{T}A\right)+C$$

subject to diag(A)=1, and A is symmetric and positive semidefinite

I is the unit matrix. Since $C=-\frac{\lambda_{2}}{2}tr\left(L^{T}I\right)=-\frac{\lambda_{2}}{2}tr\left(L\right)$ is a constant that doesn’t contain unknown variables, we conclude that the objective function and the constraints of the original problem to find single-cell compartments is the same as the regular Max-cut problem. We implement this SDP using the python package cvxpy [38] and cvxopt https://github.com/cvxopt/cvxopt, with the SCS solver [39], to compute the resulting matrix A.

### Decomposition by approximation and random projection

Since $A={VV}^{T}$, we can recover the vector matrix $V={\left(v_{1},\;v_{2},\;\ldots ,{\;v}_{n}\right)}^{T}$ from $\begin{array}{c} A \end{array}$ using Cholesky decomposition. However, Cholesky requires $\begin{array}{c} A \end{array}$ to be strictly positive semidefinite, which may not hold due to the convergence threshold in the optimization. Instead, we apply LDL decomposition on to generate a symmetric matrix $\begin{array}{c} A \end{array}$, which is very close to a positive semidefinite matrix:


$$A=UDU^{T}$$

where U is a lower unit triangular matrix, $D=diag(d_{i})$ is a diagonal matrix with diagonal entries $d_{i}$. If A is an approximately positive semidefinite matrix, D may contain some negative entries with small values. To enforce non-negativity, we set $d_{i}^{\prime }=0$ if $d_{i}<0$ and $d_{i}^{\prime }=d_{i}$ elsewhere to obtain a new diagonal matrix $D^{\prime }=diag(d_{i}^{\prime })$. We define the square root matrix of $D^{\prime }$ as:


$$\sqrt{D^{\prime }}=diag(\sqrt{d_{i}^{\prime }})$$

Since we know $D^{\prime }=\sqrt{D^{\prime }}\sqrt{D^{\prime }}=\sqrt{D^{\prime }}{\sqrt{D^{\prime }}}^{T}$, we can generate a new matrix $A^{\prime }$ by:


$$A^{\prime }=UD^{\prime }U^{T}=U\sqrt{D^{\prime }}{\sqrt{D^{\prime }}}^{T}U^{T}=(U\sqrt{D^{\prime }}){\left(U\sqrt{D^{\prime }}\right)}^{T}$$

Hence, this yields a Cholesky-like decomposition by approximating the diagonal entries of D and formulate the final vectors by:

$V^{\prime }=U\sqrt{D^{\prime }}$ so that $A^{\prime }=V^{\prime }{V^{\prime }}^{T}$

The difference between $A^{\prime }$ and A can be evaluated by Euclidean norm of their difference and reverse triangular inequality:


$${\left\|\;A^{\prime }-A\right\|}_{2}\geq \left|{\left\|\;A^{\prime }\right\|}_{2}-{\left\|\;A\right\|}_{2}\right|=\left|{\left\|V^{\prime }{V^{\prime }}^{T}\right\|}_{2}-{\left\|\;{VV}^{T}\right\|}_{2}\right|$$

According to the property of Euclidean norm, we have ${\left\|MM^{T}\right\|}_{2}={\left\|M\right\|}_{2}^{2}$ for any matrix M:


$${\left\|\;A^{\prime }-A\right\|}_{2}\geq \left|{\left\|V^{\prime }\right\|}_{2}^{2}-{\left\|V\right\|}_{2}^{2}\right|=\left|({\left\|\;V^{\prime }\right\|}_{2}+{\left\|\;V\right\|}_{2})({\left\|\;V^{\prime }\right\|}_{2}-{\left\|\;V\right\|}_{2})\right|=\left|{\left\|\;V^{\prime }\right\|}_{2}+{\left\|\;V\right\|}_{2}\right|\left|{\left\|\;V^{\prime }\right\|}_{2}-{\left\|\;V\right\|}_{2}\right|$$

While the difference between $V^{\prime }$ and V is measured by Euclidean norm of their difference and triangular inequality:


$${\left\|\;V^{\prime }-V\right\|}_{2}\leq {\left\|\;V^{\prime }\right\|}_{2}+{\left\|\;V\right\|}_{2}$$

Overall, we have the following relationship:


$${\left\|\;V^{\prime }-V\right\|}_{2}\leq \frac{\left|{\left\|\;V^{\prime }\right\|}_{2}+{\left\|\;V\right\|}_{2}\right|\left|{\left\|\;V^{\prime }\right\|}_{2}-{\left\|\;V\right\|}_{2}\right|}{\left|{\left\|\;V^{\prime }\right\|}_{2}-{\left\|\;V\right\|}_{2}\right|}\leq \frac{{\left\|\;A^{\prime }-A\right\|}_{2}}{\left|\sqrt{{\left\|\;A^{\prime }\right\|}_{2}}-\sqrt{{\left\|\;A\right\|}_{2}}\right|}=\frac{{\left\|\;E\right\|}_{2}}{\left|\sqrt{{\left\|\;A+E\right\|}_{2}}-\sqrt{{\left\|\;A\right\|}_{2}}\right|}=O(\sqrt{{\left\|\;E\right\|}_{2}})$$

where $E=A^{\prime }-A$ denotes the approximation error between $A^{\prime }$ and A. Hence, we have proven that if we can make the approximated matrix $A^{\prime }$ as close to matrix A as possible and reduce the error as much as possible, the resulting matrix $V^{\prime }$ generated from decomposition will also be very close to matrix V. Let $V^{\prime }={\left(v_{1}^{\prime },\;v_{2}^{\prime },\;\ldots ,\;v_{n}^{\prime }\right)}^{T}$, where each $v_{i}^{\prime }$ indicates each node i embedding in hyperspace. To partition nodes into two groups, we apply a random hyperplane with normal vector r, to cut the hyperspace into two sub-hyperspaces, where all vectors are divided into two groups with positive or negative dot products $S=\left\{i:{v_{i}^{\prime }}^{T}r>0\right\}$ and $\overset{―}{S}=\left\{i:{v_{i}^{\prime }}^{T}r<0\right\}$. Using the two groups to form a vector $s={\left(s_{1},\;s_{2},\;...,\;s_{N}\right)}^{T}$, where $s_{i}=1$ if i∈S and $s_{i}=-1$ if $i\in \overset{―}{S}$, we calculate the objective value of the Max-cut problem by:


$$obj={\frac{1}{4}s}^{T}Ls$$

To obtain the best cut, we choose to apply random rounding multiple times until the objective value exceeds a threshold. Goemans and Williamson prove that the approximation ratio of random projection is about 0.878 which improves the performance substantially over other approximations [26,27]. Accordingly, we set the threshold to be 0.878OPT(Max Cut), where OPT(Max Cut) indicates the optimal result.

### Transforming 3D structures to graphs

We generated a population of H1-hESC 3D genome structures with our integrative genome modeling (IGM) platform [25] using data from Hi-C [3], DamID [33] and SPRITE [34] experiments. We generated 1,000 diploid whole-genome structures containing 3D coordinates of 30,332 200 kb regions in each cell. Each chromosome structure in each cell is then embedded as a graph with each chromatin region as a node, and edges connecting nodes with weights derived from a combination of the spatial distance between two genomic regions and their distances from nuclear bodies, capturing aspects of nuclear architecture.

The compartment prediction depends on how we construct these graphs from 3D genome structures by choosing proper edge weights. Firstly, the graph is undirected. Secondly, since two chromatin regions with closer spatial distance have generally higher contact probabilities, we assign smaller edge weights between nodes if their spatial distances in 3D are smaller than than expected 3D distance by their sequence distance alone. Varoquaux et al [40] demonstrates that the expected spatial distance $\left(e_{ij}\right)$ between two regions i, j and the difference in their sequence position $\left|g_{i}-g_{j}\right|$ follows $e_{ij}\sim {\left(\left|g_{i}-g_{j}\right|\right)}^{\frac{1}{2}}$ at small ranges, with $g_{i}$, $g_{j}$ as the sequence position of chromatin regions *i* and *j* in a chromosome.

The observed spatial distances $(d_{ij}\;\$(d_{ij}\;)\;$ between pairs of chromatin regions in a genome structure are calculated using the Euclidean distance between their 3D coordinates:


$$d_{ij}={\left\|x_{i}-x_{j}\right\|}_{2}=\sqrt{{\left(x_{i1}-x_{j1}\right)}^{2}+{\left(x_{i2}-x_{j2}\right)}^{2}+{\left(x_{i3}-x_{j3}\right)}^{2}}$$

where $\left(x_{i1},x_{i2},x_{i3}\right)$ and $\left(x_{j1},x_{j2},\;x_{j3}\right)$ are 3D coordinates of region i and region j. 3D coordinates are obtained from our genome structure modeling or derived from multiplexed FISH imaging (e.g., DNA MERFISH or SeqFISH+ experiments).

We consider the ratio of observed spatial distance against expected spatial distance $\frac{observed\;spatial\;distance}{expected\;spatial\;distance}=\;\frac{d_{ij}}{e_{ij}}=\frac{{\left\|x_{i}-x_{j}\right\|}_{2}}{{\left(\left|g_{i}-g_{j}\right|\right)}^{\frac{1}{2}}}$ as part of the edge weight so that chromatin regions at longer distances than expected are more likely to be classified into different compartments.

Nuclear speckles act as hubs for pre-mRNA processing and highly transcribed genes are often located in close proximity to speckles. Thus, distances to nuclear speckles can act as potential indicators for active chromatin compartments. Hence, we incorporate a scaling factor $\left|z_{i}-z_{j}\right|$ to define edge weights that contribute the similarity of two chromatin regions i and j with respect to their distances to their closest nuclear speckles. $z_{i}$ and $z_{j}$ define the z-score normalized distances between chromatin regions *i* and *j* and their nearest nuclear speckles. Therefore, edge weights between two chromatin regions are further normalized by the similarity of their speckle affinity.

The distance between a chromatin region and its nearest speckle is obtained from the genome structure models or from DNA-MERFISH datasets (3 Mb resolution) [7].

Structures from SeqFISH+ imaging datasets (1 Mb resolution) [8] provide only intensities of imaged speckle marker antibodies at locations of certain loci. We estimated the speckle distances by assuming speckle intensity decays at a quadratic rate:


$$z_{i}=\frac{1}{\sqrt{s_{i}}}$$

where $s_{i}$ is the imaged speckle intensity at loci i.

The weight $w_{ij}$ of an edge between nodes i and node j is then defined by the product of the following factors:


$$w_{ij}=\frac{{\left|z_{i}-z_{j}|\|x_{i}-x_{j}\right\|}_{2}}{{\left(\left|g_{i}-g_{j}\right|\right)}^{\frac{1}{2}}}$$

where $x_{i}$ and $x_{j}$ are the 3D coordinates, $g_{i}$ and $g_{j}$ are the genomic positions, $z_{i}$ and $z_{j}$ are the z-scores of speckle distances of chromatin regions i and j.

Because compartments are more likely to be determined by local structural relationships, edges between nodes i and node j are removed when their distance is above a threshold, specifically when ${\left\|x_{i}-x_{j}\right\|}_{2}>16R_{bead}$, where $R_{bead}$ is the excluded volume of a sphere representing chromatin regions of 200kb sequence length. For DNA-MERFISH and SeqFISH+ structures, we set $R_{bead}=100\;nm$. Finally, min-max normalization is applied to the edge weights so that all weights range between 0 and 1:


$$w_{ij}^{\prime }=\frac{w_{ij}}{\max_{k,\;l}\;w_{kl}\;}$$

The matrix $W=(w_{ij}^{\prime })$ will be the final adjacency matrix of the graph, which is further used to generate the Laplacian matrix of the graph.

Due to the large size of the graph and the long running time, we select 500 copies of each studied chromosome from our structure population to perform the analysis.

### Prediction of compartments

We apply our algorithm to each chromosome structure graph individually to calculate compartments for every single-cell structure. Given two sets nodes sets, S and $\overset{―}{S}$, generated by the MaxComp algorithm, we set the active compartment $S_{a}=S$ and inactive compartment $S_{b}=\;\overset{―}{S}$ if the average speckle distance of all nodes in S is smaller than that of $\overset{―}{S}$; otherwise $S_{a}=\overset{―}{S}$ and $S_{b}=$ S.

### Compartment profile vector

A compartment profile vector $c_{k}={\left(c_{k1},\;c_{k2},\;...,\;c_{kN}\right)}^{T}$ for a given chromosome structure k is calculated based on the prediction $S_{a}$ and $S_{b}$. We set $c_{ki}=1$ if region i in structure k is assigned to the **A** compartment otherwise $c_{ki}=0$.

### Ensemble compartment vector

We define the ensemble compartment vector as $c^{ens}=\frac{1}{K}\sum_{k=1}^{K}c_{k}={\left(c_{1}^{ens},\;c_{2}^{ens},\;...,\;c_{N}^{ens}\right)}^{T}$, where each element $c_{i}^{ens}$ represents the fraction of times in the population where region i is assigned to the **A** compartment. The ensemble compartment vector is therefore the sum of all compartment profile vectors across all chromosome structures divided by the total number of structures.

### Compartment frequency

The compartment frequency $f_{i}$ of a genomic region i is defined as:


$$f_{i}=c_{i}^{ens}\;-I_{\;c_{i}^{ens}<\;0.5}$$

where $I_{\;c_{i}^{ens}<\;0.5}$ is an indicator function equal to 1 if $c_{i}^{ens}<\;0.5$, and 0 otherwise. The absolute value of $\left|f_{i}\right|$ describes the fraction of times a genomic region i is predicted to be in its majority compartment across all structures. Positive values indicate the compartment frequency of region i in compartment **A**, when the region is in compartment **A** in most of the structures. Negative values indicate the absolute value of compartment frequency of region i in compartment **B**, when the region is in the **B** compartment in the majority of structures. Subsequently, the compartment frequency profile for a chromosome is defined as:


$$f={\left(f_{1},\;f_{2},\;...,\;f_{N}\right)}^{T}$$

This vector can be directly compared to the PC1 profile from ensemble Hi-C data.

### Normalized compartment frequency

The normalized compartment frequency $f_{i}^{norm}$ of a genomic region i is defined as:


$$f_{i}^{norm}=c_{i}^{ens}\;-0.5$$

The normalized compartment frequency is used to calculate the correlation with PC1 values derived from ensemble Hi-C-based compartment predictions.

### Compartment variability

The compartment variability ${\delta c}_{i}$ for chromatin region i is calculated as the standard deviation of its compartment profile value across all K structures of the population:


$${\delta c}_{i}=\sqrt{\frac{\sum_{k=1}^{K}{\left(c_{ki}-\overset{―}{c_{i}}\right)}^{2}}{K}}$$

where $c_{ki}$ is either 1 indicating that region i is in compartment **A** in structure k or 0 indicating that the same region i is in compartment **B**. $\overset{―}{c_{i}}$ is the average value of $c_{i}$ of region i across all K structures: $\overset{―}{c_{i}}=\frac{1}{K}\sum_{k=1}^{K}c_{ki}$. The larger the value of ${\delta c}_{i}$ is, the more variable the compartment annotations of region i are across the population of structures.

### Structural features

#### Radial position (RAD).

The radial position of a chromatin region i in structure s in a spherical nucleus is calculated as:


$$r_{i}^{\left(s\right)}=\frac{{\left\|x_{is}\right\|}_{2}}{R_{nuc}}$$

where $x_{is}$ is the the 3D coordinates of bead i in structure *s*, and $R_{nuc}=5\;\mu m$ is the nucleus radius. $r_{i}^{\left(s\right)}=0$ indicates the region i is at the nuclear center while $r_{i}^{\left(s\right)}=1$ means it is at the nuclear surface. The radial position variability (δRAD) of region i in the population is calculated as:


$${\Delta r}_{i}=\log_{2}\frac{\sigma_{i}}{\overset{―}{\sigma }\;}$$

where $\sigma_{i}$ is the standard deviation of the population of radial positions of region i and $\overset{―}{\sigma }$ is the mean standard deviation calculated from all regions within the same chromosome of the target region.

#### Radius of gyration (RG).

The local compaction of the chromatin fiber at the location of a given locus is estimated by the radius of gyration for a 1 Mb region centered at the locus. To estimate the values along an entire chromosome we use a sliding window approach over all chromatin regions in a chromosome. The radius of gyration for a 1 Mb region centered at locus i in structure s, is calculated as:


$$g_{i}^{\left(s\right)}=\sqrt{\frac{1}{5}\sum_{j=1}^{5}d_{j}^{2}}$$

where $d_{j}$ is the distance between the chromatin region j to the center of mass of the 1-Mb region.

#### Distances to nuclear bodies.

Distances for each chromatin region i to various nuclear bodies—including speckle distance (SpD) and lamina distance (LmD)—are calculated by measuring the distance between the surface of each chromatin region i to the nearest speckle and lamina [30]. In each 3D genome structure model, speckle locations are estimated by the geometric centers of highly connected interaction subgraphs of chromatin regions with the top 10% SON TSA-seq signals following a procedure described in [30], while the locations of lamina is identified as the nuclear boundary. For DNA MERFISH data, SpD and LmD are directly obtained from the datasets [7]. The speckle variability (δSpD) of region i is calculated as the standard deviation of its speckle distances across the population of structures.

#### Interchromosomal contacts.

The calculation of inter-chromosomal contacts is similar to the calculation of contact frequency matrix but is based on a larger contact range. For a given 200kb region, its interchromosomal contacts is the total number of contacts with any target inter-chromosomal regions from the same genome structure within range $R_{soft}=1,000\;nm$.

### Compartmentalization score

We define the contact-compartmentalization score (CCS), denoted as $s_{c}$, as the ratio of total intra-compartment contacts to all contacts in a structure (intra-compartment contacts + inter-compartment contacts):


$$s_{c}=\frac{n_{intra}}{n_{intra}+n_{inter}}$$

where $n_{intra}$ and $n_{inter}$ are the numbers of unique intra- and inter-compartment contacts, respectively. A contact is defined when the spatial distance between two regions is less than or equal to $3R_{bead}$ for modelled structures and SeqFISH+ coordinates [8] and $4R_{bead}$ for DNA-MERFISH coordinates [7], accounting for different locus resolutions.

The CCS score allows comparison of different compartment annotations (e.g., from MaxComp or other methods). A higher CCS indicates stronger intra-compartment connectivity and thus a more accurate partition.

Similarly, we define the distance-compartmentalization score (DCS), denoted $s_{d}$, as:


$$s_{d}=\frac{d_{intra}}{d_{intra}+d_{inter}}$$

where $d_{intra}$ and $d_{inter}$ are the sums of intra- and inter-compartment distances, respectively. A lower $s_{d}$ indicates more compact intra-compartment regions compared to inter-compartment ones, reflecting better chromatin subcompartment segregation.

### Preprocessing of imaging tracing datasets

For the imaging dataset, 7,000 DNA-MERFISH copies of chromosome 6 and chromosome 10 from the IMR90 cell line are obtained from Su et al [7] together with their corresponding speckle distances, lamina distances, nucleoli distances and transcription profiles with transcription on or off (nascent transcript imaged or not) for genes measured by RNA MERFISH. All datasets are preprocessed by linear interpolation to remove missing values. Structural information of DNAseqFISH+ including coordinates, speckle densities and transcription information containing the number of detected spots corresponding to mRNA transcript for more than 40 genes from 444 cells (888 copies) of the mESC cell line are obtained from Takei et al [8]. We preprocess the datasets to generate reasonable speckle locations for each cell using experimental SON TSA-seq following an approach described in [30]. Similarly, all datasets are preprocessed by linear interpolation to remove missing values. To avoid the impact of zero transcription, we remove the bottom 5% cells in the number of transcription spots for each gene when performing transcription ratio and paired change analysis. The genes are mapped to genomic regions nearest to their promoters in the reference genome [41]. For each gene, we first divide the cells into different groups, where **A** cells (**B** cells) indicate the corresponding locus on both copies is predicted to be in the compartment **A** (**B**). Then its transcription ratio trans is calculated by the log fold change of the average transcription level (number of mRNA transcript spots detected) in **A** cells $t_{A}$ over the average transcription level in **B** cells $t_{B}$:


$$trans=\log_{2}(\frac{t_{A}}{t_{B}})$$

Normalized $t_{A}$ and $t_{B}$ are calculated in the same way for each gene after min-max normalization on the population-wide transcription levels to conduct paired comparison with paired t-test. To compare with gene density, we calculate the total number of genes from the UCSC Genome Browser RefSeq [41] are located within each imaged loci and construct a gene density profile for each studied chromosome.

### Hi-C-based PCA

The Hi-C-based PCA profile $c_{\exp}$ are obtained from the in-situ Hi-C dataset for H1-hESC (4DNESX75DD7R) [3], which are directly calculated by the largest principal component (the eigenvector corresponds to the largest eigenvalue) of the covariance matrix from the experimental ensemble Hi-C. For comparison, the PC1 values are mapped and averaged with regards to the nearest 200kb bins from the model. We measure the Pearson’s correlation coefficient $r(c,\;c_{\exp})$ between the non-zero values from the predicted normalized compartment frequency vector c and the experimental profile $c_{\exp}$ for each studied chromosome. The Hi-C-based PCA profile $c_{\exp}$ are obtained from Rao et al for IMR90 (4DNESSM1H92K) [2] and Takei et al [8] for mESC. Similarly, we use the nearest PC1 values for each imaged loci for comparison with predicted profiles.

### Distance-based PCA

Principal component analysis is mathematically performed by eigenvector decomposition on the input matrix, which can not only be applied on contact matrices, but also be adapted to pairwise distance analysis. The approach has been previously used by imaging related studies such as Wang et al [4] and Sawh et al [35]. We first normalize the mean distance matrix by pairwise genomic distances through fitted power-law function, and then calculate the pairwise Pearson’s correlation matrix between every row and column pair. Using it as the covariance matrix for PCA analysis, the resulting vector corresponding to the largest principal component with positive and negative entries can be used to generate compartments that are comparable with annotations from other methods.

### CG phasing

Another frequently used approach is phasing by genomic information such as CpG density or CG content [22], where we have prior knowledge that high CG contents correspond to active compartments. We obtain CG contents for both hg38 and mm10 reference genomes from the UCSC genome browser [41]. For each locus, we calculate the mean CG contents from all loci (including itself) within its neighborhood (250 nm). The resulting vector measures the smoothed CG contents at single-cell levels, where the higher the value is the more likely the chromatin region belongs to **A** compartment. Eventually we may calculate the log fold change against the average to get **A**/**B** annotations by positive or negative signs as what we have explained in the MaxComp prediction.

### Structure visualization

All chromosome structures together with nuclear envelopes and speckles are visualized by UCSF Chimera [42].

### Computational requirements

The time and memory requirements for MaxComp depends on the population size and the chromosome size. For a modeled human chromosome 6 which is 171 Mb and at 200 kb resolution forms an input graph with 855 nodes, the algorithm takes on average 30–40 minutes to generate a single-cell compartment profile. In total less than 50GB memory is required to calculate and store 500 single-cell compartment profiles. When using the imaging dataset at 3 Mb resolution which forms an input graph with 55 nodes, the running time reduces to less than 1 minute. For the other chromosomes, the fewer the chromatin regions are, the shorter time and less memory MaxComp will require.

For IGM modeling, the generated population-based models consist of 1,000 genome copies from Boninsegna et al. [2] requires between 10–15 hours of parallel computation using 250 nodes, with a total memory of 4 GB for the controller and 2 GB per processor. The details about IGM requirements are available at https://github.com/alberlab/igm/.

## Supporting information

S1 Fig Correlations between average distances and normalized genomic distances (A) Scatter plots of average distances from DNA-MERFISH Chr6 and Chr10 [7] against the corresponding normalized genomic distances showed together with the fitted quadratic curves and the Pearson’s correlation coefficients. (B) Scatter plots of average distances from SeqFISH+ Chr5 and Chr15 [8] against the corresponding normalized genomic distances showed together with the fitted quadratic curves and the Pearson’s correlation coefficients.
(TIF)

S2 Fig Comparison between population sizes on predicted profiles and their correlations (A) The experimental profile obtained from principal component analysis on ensemble Hi-C matrix. (B) The predicted normalized compartment frequencies by MaxComp on populations of modeled Chr6 structures with different sizes. (C) Scatter plots between PC1 values from the Hi-C-based PCA and various predicted compartment profiles showed with Pearson’s correlation coefficients. We observe increased value in the coefficient as population size grows larger.
(TIF)

S3 Fig Prediction of model compartments by MaxComp and its comparison with the ground truth on H1-hESC Chr15 and Chr18 (A) The experimental profile obtained from the Hi-C-based principal component analysis and the compartment profile predicted by MaxComp of 500 modeled structures of Chr15. (B) The experimental profile obtained from the Hi-C-based principal component analysis and the compartment profile predicted by MaxComp of 500 modeled structures of Chr18. (C) Scatter plot between the normalized compartment frequencies of MaxComp and the PC1 values showed together with the Pearson’s correlation coefficient between the two samples on each chromosome. (D) Comparison of speckle distances (p-value = 1.84e-153 and 9.64e-102), lamina distances (p-value = 1.03e-27 and 2.35e-56), radial positions (p-value = 1.03e-27 and 2.35e-56), radius of gyration (p-value = 9.54e-41 and 2.25e-54) and interchromosomal contacts (p-value = 1.89e-48 and 2.55e-76) between compartment **A** beads and compartment **B** beads on the population of structures of Chr15 and Chr18.
(TIF)

S4 Fig Prediction of model compartments by MaxComp and its comparison with the ground truth on H1-hESC Chr8 and Chr12 (A) The experimental profile obtained from the Hi-C-based principal component analysis and the compartment profile predicted by MaxComp of 500 modeled structures of Chr8. (B) The experimental profile obtained from the Hi-C-based principal component analysis and the compartment profile predicted by MaxComp of 500 modeled structures of Chr12. (C) Scatter plot between the normalized compartment frequencies of MaxComp and the PC1 values together with the Pearson’s correlation coefficient between the two samples on each chromosome. (D) Comparison of speckle distances (p-value = 8.61e-70 and 5.32e-121), lamina distances (p-value = 8.53e-46 and 1.13e-35), radial position (p-value = 8.53e-46 and 1.13e-35), radius of gyration (p-value = 2.70e-52 and 2.61e-63) and interchromosomal contacts (p-value = 4.81e-68 and 1.37e-61) between compartment **A** beads and compartment **B** beads on the population of structures of Chr8 and Chr12.
(TIF)

S5 Fig Selected example of model compartments predicted by MaxComp for the whole genome of H1-hESC (A) The predicted compartments for each chromosome copy from structure 0 of H1-hESC. (B) Selected chromosomes from structure 0 showed together with the envelope indicates compartment **A** and compartment **B** are segregated within the envelope. The section through the genome shows compartment **A** and compartment **B** are clustered with each other in the inferior region. Predicted speckles are basically associated with compartment **A** beads rather than compartment **B** beads.
(TIF)

S6 Fig Transcription analysis on single-cell DNA-MERFISH Chr6 compartments predicted by MaxComp (A) Selected examples with more than or equal to 8 locus with active transcriptions of compartment prediction and transcription signals on DNA MERFISH structures [7] (Red bars indicate compartment **A**, blue bars represent compartment **B** while gray bars are where transcription is on (nascent transcript is imaged)). (B) Comparison between the gene density from RefSeq, the transcription frequency from DNA MERFISH and the compartment profile predicted by MaxComp. (C) Scatter plots between gene density, transcription frequency and predicted compartment profile showed with Spearman’s correlation coefficients.
(TIF)

S7 Fig Transcription analysis on single-cell DNA-MERFISH Chr10 compartments predicted by MaxComp (A) Selected examples with more than or equal to 10 locus with active transcriptions of compartment prediction and transcription signals on DNA MERFISH structures [7] (Red bars indicate compartment **A**, blue bars represent compartment **B** while gray bars are where transcription is on (nascent transcript is imaged)). (B) Comparison between the gene density from RefSeq, the transcription frequency from DNA MERFISH and the compartment profile predicted by MaxComp. (C) Scatter plots between gene density, transcription frequency and predicted compartment profile showed with Spearman’s correlation coefficients.
(TIF)

S8 Fig Comparison between distributions of normalized transcription levels from SeqFISH+ Comparison of normalized transcription levels (numbers of mRNA transcript spots detected) between A cells and B cells for genes measured by SeqFISH+ [8] showed together with the paired t-test We find most of the genes have increased transcription levels when shifting from state **B** to state **A** (showed in brown).
(TIF)
