# Supplementary figures and images for: MaxComp: Predicting single-cell chromatin compartments from 3D chromosome structures
Source: PLoS Comput Biol. 2025 May 23;21(5):e1013114. doi: 10.1371/journal.pcbi.1013114 (PMC12133197; doi:10.1371/journal.pcbi.1013114)

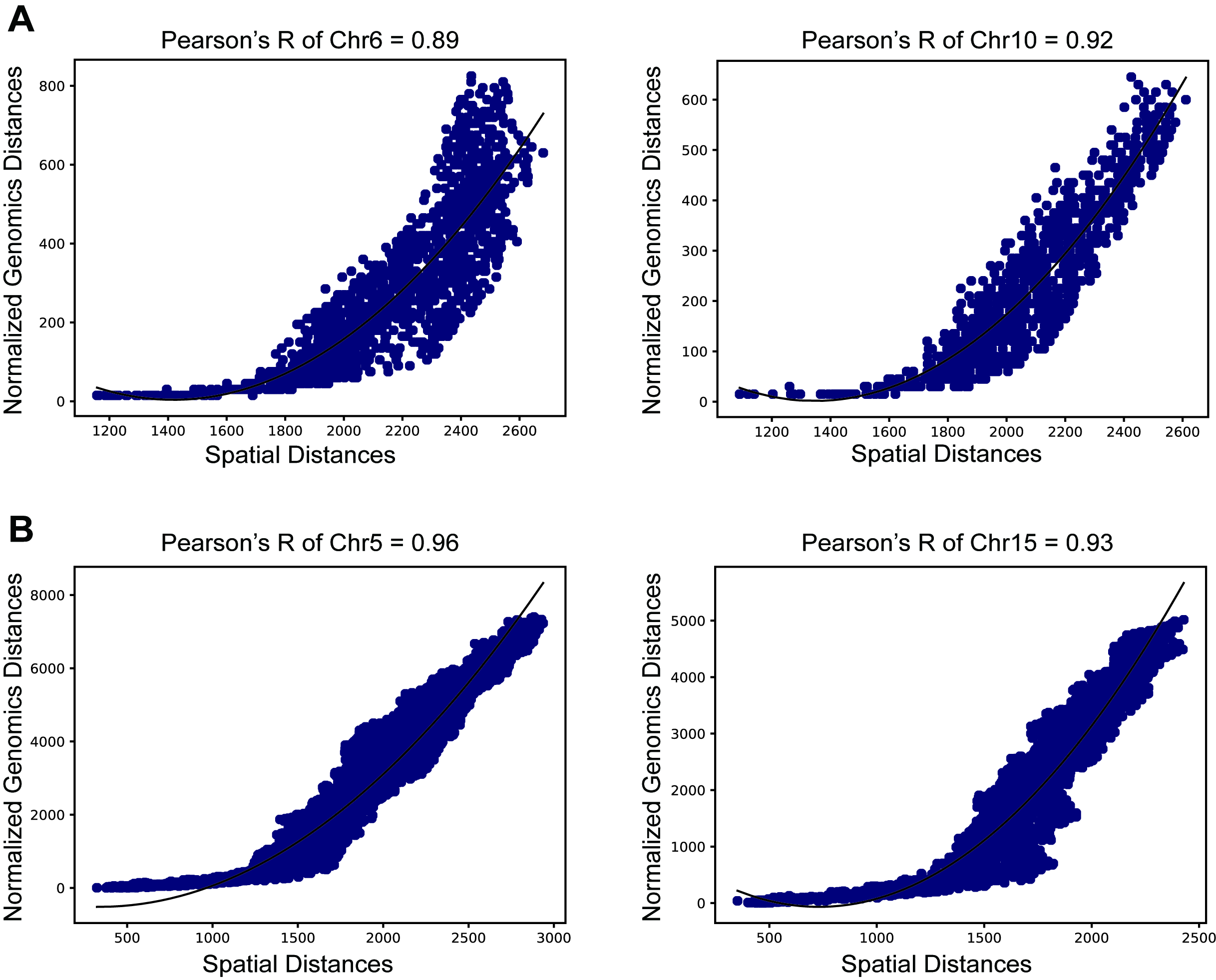

Supplement: S1 Fig — (A) Scatter plots of average distances from DNA-MERFISH Chr6 and Chr10 [7] against the corresponding normalized genomic distances showed together with the fitted quadratic curves and the Pearson’s correlation coefficients. (B) Scatter plots of average distances from SeqFISH+ Chr5 and Chr15 [8] against the corresponding normalized genomic distances showed together with the fitted quadratic curves and the Pearson’s correlation coefficients. (TIF) [file pcbi.1013114.s001.tif]

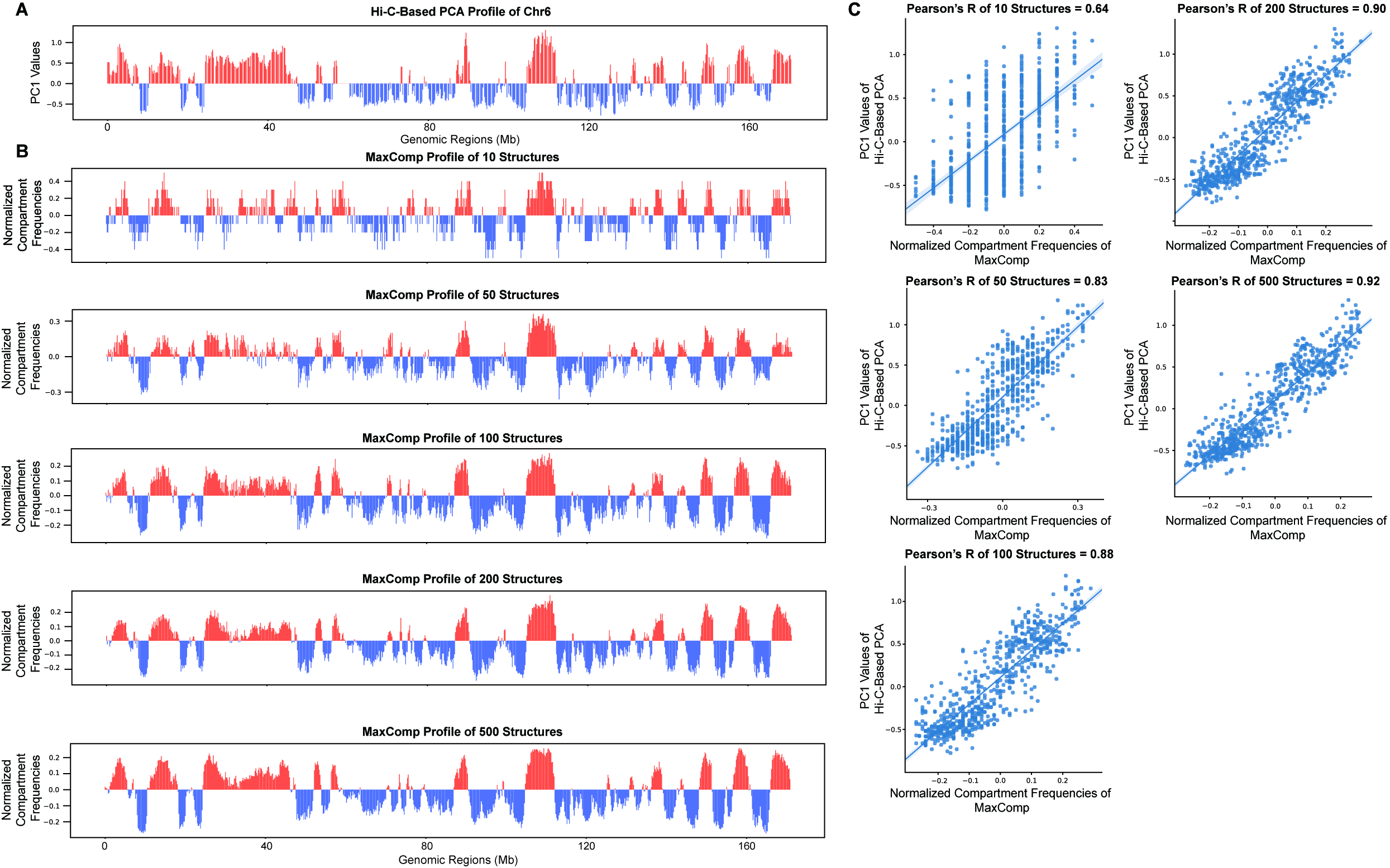

Supplement: S2 Fig — (A) The experimental profile obtained from principal component analysis on ensemble Hi-C matrix. (B) The predicted normalized compartment frequencies by MaxComp on populations of modeled Chr6 structures with different sizes. (C) Scatter plots between PC1 values from the Hi-C-based PCA and various predicted compartment profiles showed with Pearson’s correlation coefficients. We observe increased value in the coefficient as population size grows larger. (TIF) [file pcbi.1013114.s002.tif]

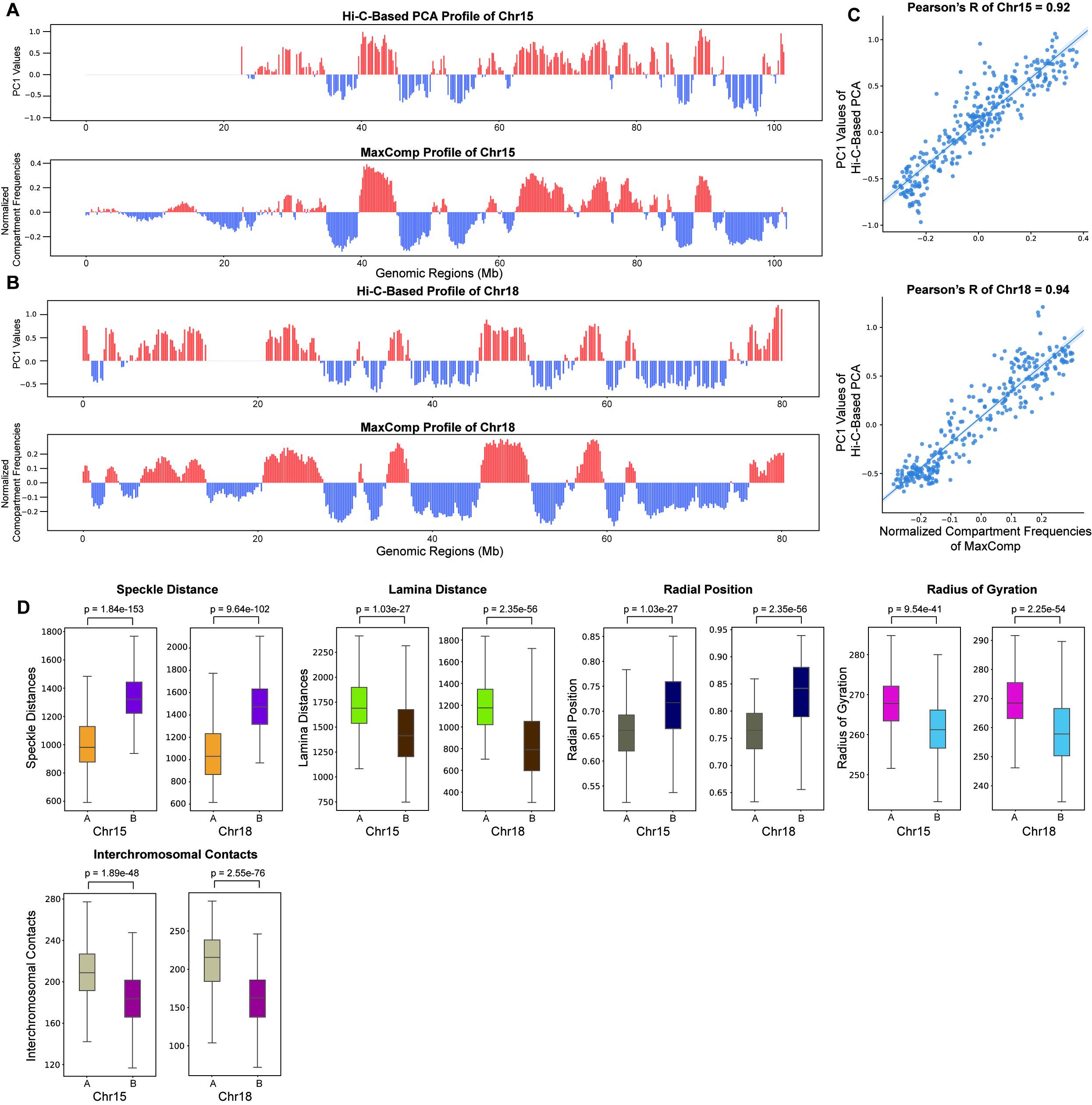

Supplement: S3 Fig — (A) The experimental profile obtained from the Hi-C-based principal component analysis and the compartment profile predicted by MaxComp of 500 modeled structures of Chr15. (B) The experimental profile obtained from the Hi-C-based principal component analysis and the compartment profile predicted by MaxComp of 500 modeled structures of Chr18. (C) Scatter plot between the normalized compartment frequencies of MaxComp and the PC1 values showed together with the Pearson’s correlation coefficient between the two samples on each chromosome. (D) Comparison of speckle distances (p-value = 1.84e-153 and 9.64e-102), lamina distances (p-value = 1.03e-27 and 2.35e-56), radial positions (p-value = 1.03e-27 and 2.35e-56), radius of gyration (p-value = 9.54e-41 and 2.25e-54) and interchromosomal contacts (p-value = 1.89e-48 and 2.55e-76) between compartment A beads and compartment B beads on the population of structures of Chr15 and Chr18. (TIF) [file pcbi.1013114.s003.tif]

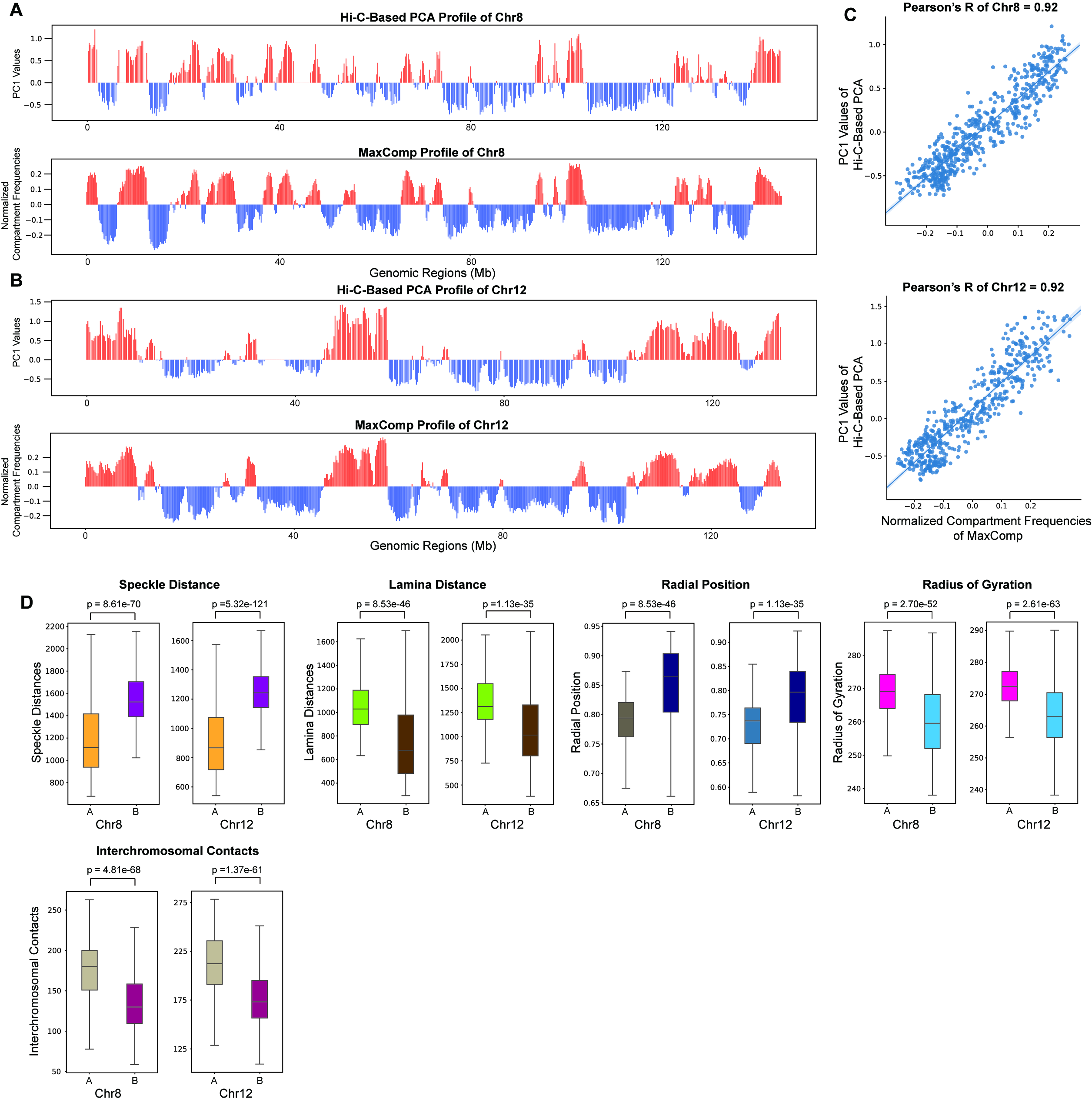

Supplement: S4 Fig — (A) The experimental profile obtained from the Hi-C-based principal component analysis and the compartment profile predicted by MaxComp of 500 modeled structures of Chr8. (B) The experimental profile obtained from the Hi-C-based principal component analysis and the compartment profile predicted by MaxComp of 500 modeled structures of Chr12. (C) Scatter plot between the normalized compartment frequencies of MaxComp and the PC1 values together with the Pearson’s correlation coefficient between the two samples on each chromosome. (D) Comparison of speckle distances (p-value = 8.61e-70 and 5.32e-121), lamina distances (p-value = 8.53e-46 and 1.13e-35), radial position (p-value = 8.53e-46 and 1.13e-35), radius of gyration (p-value = 2.70e-52 and 2.61e-63) and interchromosomal contacts (p-value = 4.81e-68 and 1.37e-61) between compartment A beads and compartment B beads on the population of structures of Chr8 and Chr12. (TIF) [file pcbi.1013114.s004.tif]

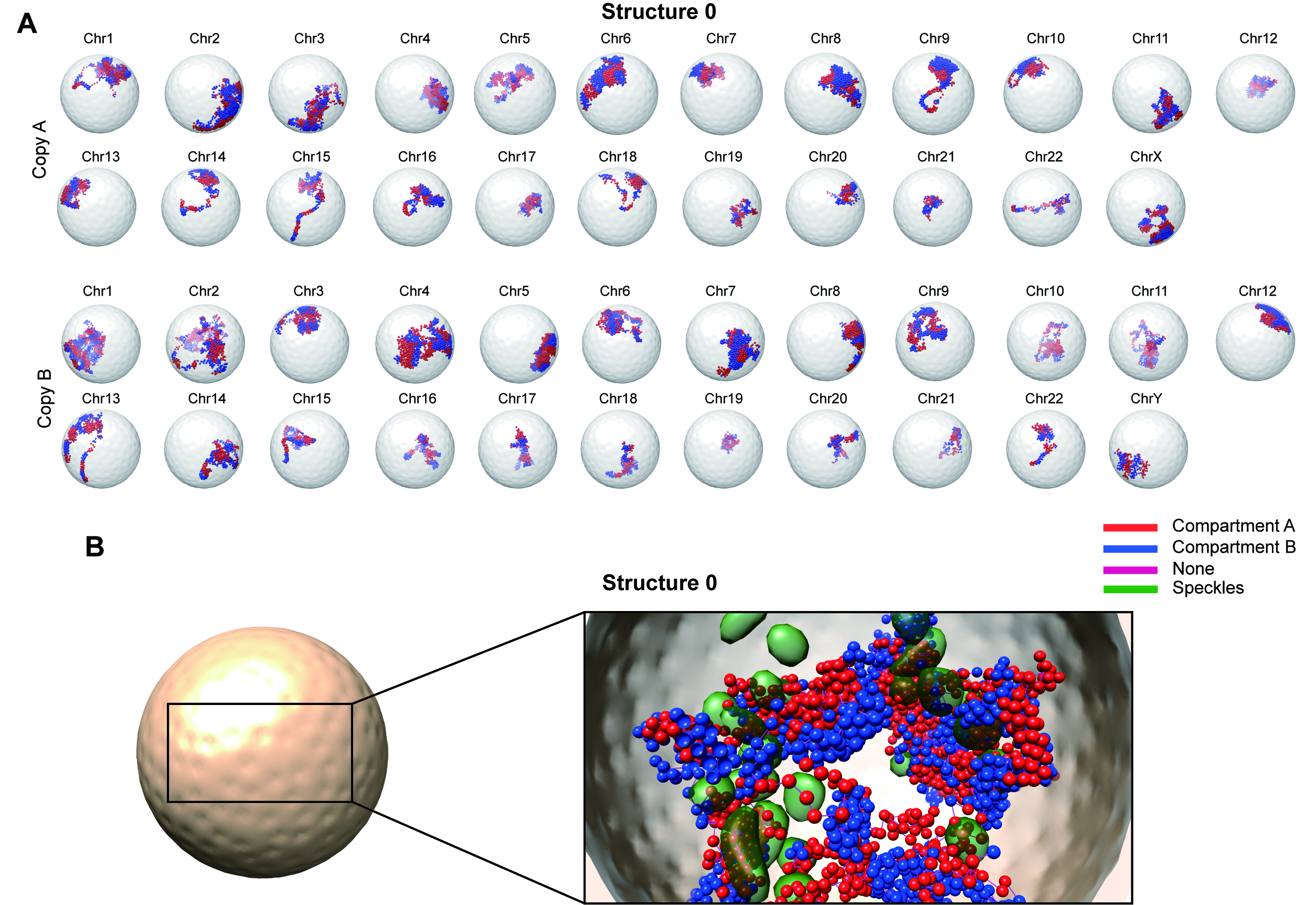

Supplement: S5 Fig — (A) The predicted compartments for each chromosome copy from structure 0 of H1-hESC. (B) Selected chromosomes from structure 0 showed together with the envelope indicates compartment A and compartment B are segregated within the envelope. The section through the genome shows compartment A and compartment B are clustered with each other in the inferior region. Predicted speckles are basically associated with compartment A beads rather than compartment B beads. (TIF) [file pcbi.1013114.s005.tif]

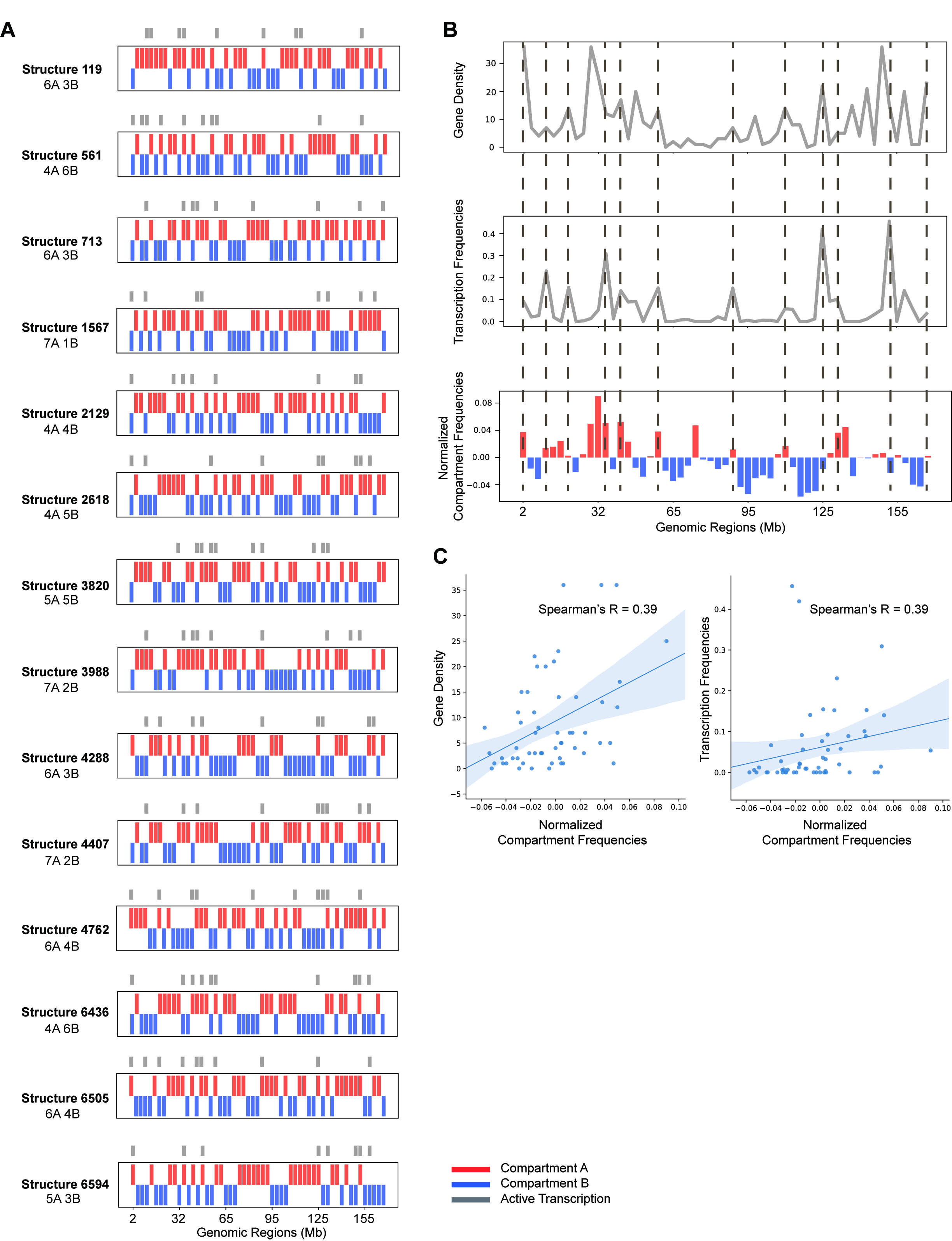

Supplement: S6 Fig — (A) Selected examples with more than or equal to 8 locus with active transcriptions of compartment prediction and transcription signals on DNA MERFISH structures [7] (Red bars indicate compartment A, blue bars represent compartment B while gray bars are where transcription is on (nascent transcript is imaged)). (B) Comparison between the gene density from RefSeq, the transcription frequency from DNA MERFISH and the compartment profile predicted by MaxComp. (C) Scatter plots between gene density, transcription frequency and predicted compartment profile showed with Spearman’s correlation coefficients. (TIF) [file pcbi.1013114.s006.tif]

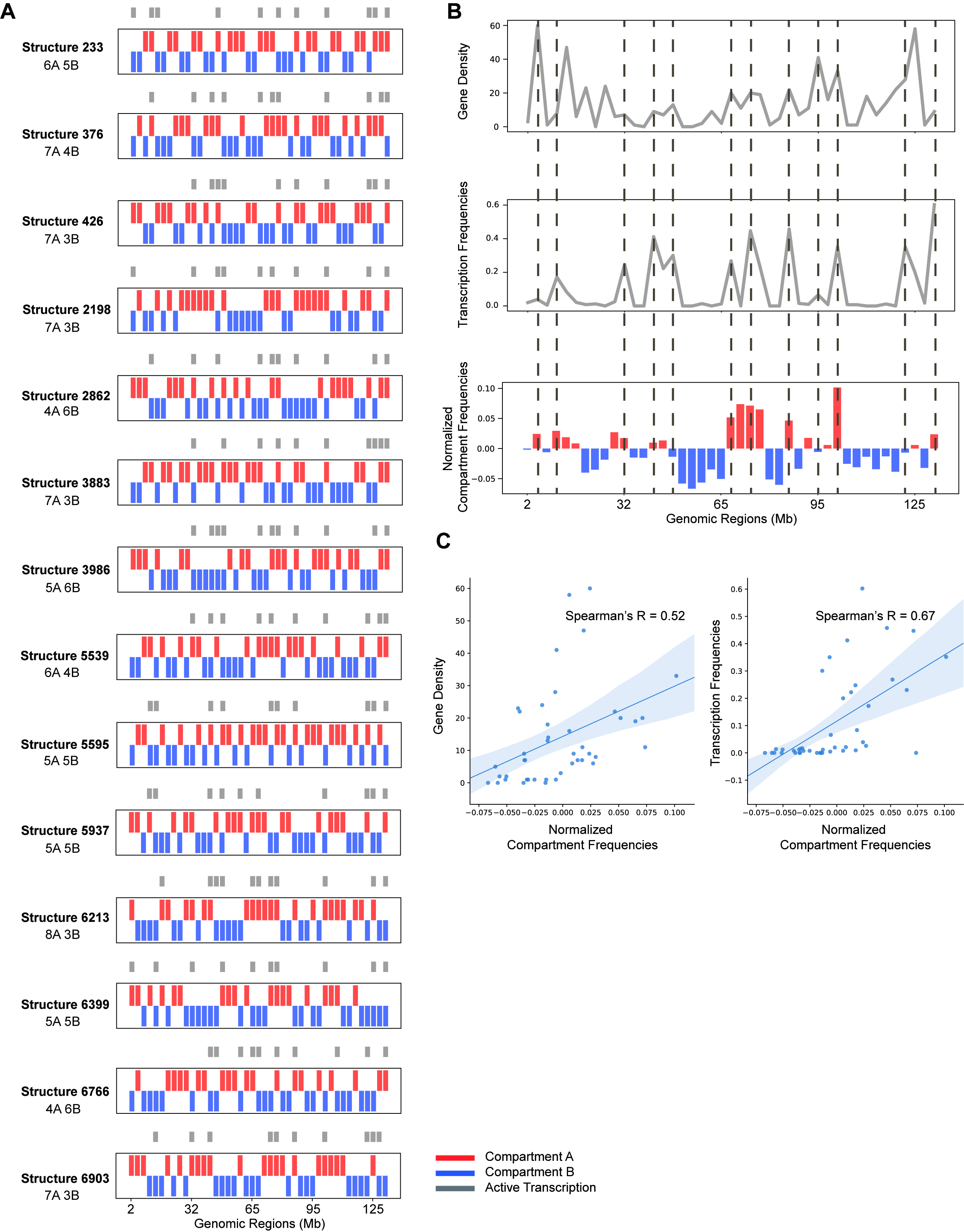

Supplement: S7 Fig — (A) Selected examples with more than or equal to 10 locus with active transcriptions of compartment prediction and transcription signals on DNA MERFISH structures [7] (Red bars indicate compartment A, blue bars represent compartment B while gray bars are where transcription is on (nascent transcript is imaged)). (B) Comparison between the gene density from RefSeq, the transcription frequency from DNA MERFISH and the compartment profile predicted by MaxComp. (C) Scatter plots between gene density, transcription frequency and predicted compartment profile showed with Spearman’s correlation coefficients. (TIF) [file pcbi.1013114.s007.tif]

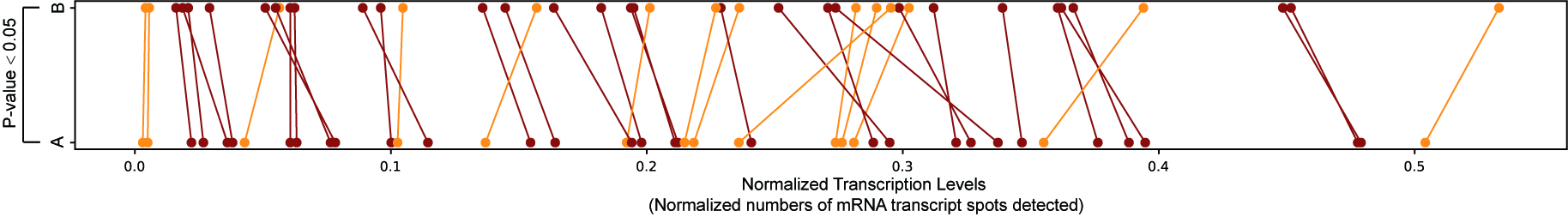

Supplement: S8 Fig — We find most of the genes have increased transcription levels when shifting from state B to state A (showed in brown). (TIF) [file pcbi.1013114.s008.tif]
